# Heterometallic Molecular and Ionic Isomers

**DOI:** 10.1021/acs.inorgchem.4c03849

**Published:** 2024-10-04

**Authors:** Yuxuan Zhang, Zheng Wei, Haixiang Han, Joyce Chang, Samantha Stegman, Tieyan Chang, Yu-Sheng Chen, John F. Berry, Evgeny V. Dikarev

**Affiliations:** † Department of Chemistry, University at Albany, Albany, New York 12222, United States; ‡ School of Materials Science and Engineering, Tongji University, Shanghai 201804, China; § Department of Chemistry, University of Wisconsin, Madison, Wisconsin 53706, United States; ∥ NSF’s ChemMatCars, Center for Advanced Radiation Source, The University of Chicago, Argonne, Chicago, Illinois 60439, United States

## Abstract

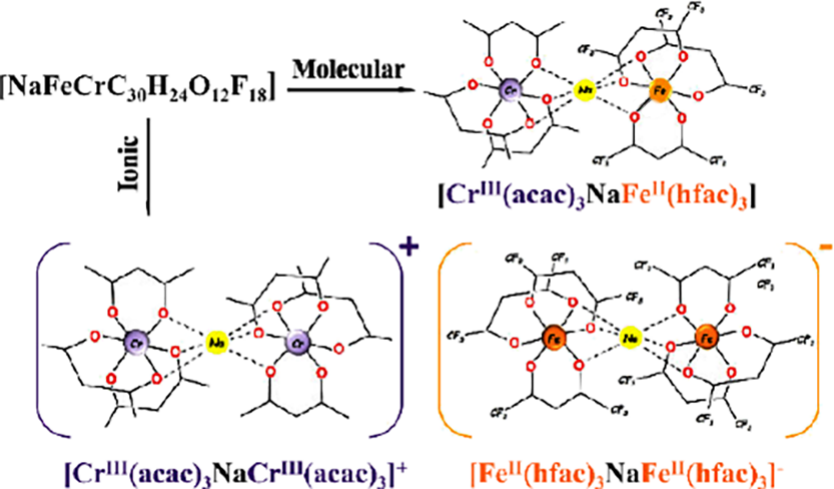

Numerous descriptions of structural isomerism in metal complexes do not list any molecular vs ionic isomers. At the same time, one of the most striking examples of structural isomerism in organic chemistry is molecular urea, which has the same atomic composition as the chemically distinct ionic ammonium cyanate. This iconic organic couple now meets its inorganic heterometallic counterpart. We introduce a new class of structural isomers, molecular vs ionic, that can be consummated in complex and coordinatively unsaturated polynuclear/heterometallic compounds. We report inorganic molecular and ionic isomers of the composition [NaCrFe (acac)_3_(hfac)_3_] (acac = acetylacetonate; hfac = hexafluoroacetylacetonate). Heterometallic molecular [Cr^III^(acac)_3_-Na-Fe^II^(hfac)_3_] (**1m**) and ionic {[Cr^III^(acac)_3_-Na-Cr^III^(acac)_3_]^+^[Fe^II^(hfac)_3_-Na-Fe^II^(hfac)_3_]^−^} (**1i**) isomers have been isolated in pure form and characterized. While both ions are hetero*bi*metallic trinuclear entities, the neutral counterpart is a hetero*tri*metallic trinuclear molecule. The two isomers exhibit distinctly different characteristics in terms of solubility, volatility, mass spectrometry ionization, and thermal behavior. Unambiguous assignment of the positions and oxidation/spin states of the Periodic Table neighbors, Fe and Cr, in both isomers have been made by a combination of characterization techniques that include synchrotron X-ray resonant diffraction, synchrotron X-ray fluorescence spectroscopy, Mössbauer spectroscopy, and DART mass spectrometry. The transformation between the two isomers that does take place in solutions of noncoordinating solvents has also been tested.

## Abstract 2

A pair of heterometallic coordination isomers, molecular [Cr^III^(acac)_3_-Na-Fe^II^(hfac)_3_] (**1m**) and ionic {[Cr^III^(acac)_3_-Na-Cr^III^(acac)_3_]^+^[Fe^II^(hfac)_3_-Na-Fe^II^(hfac)_3_]^−^} (**1i**), have been isolated in pure form and characterized. Two isomers exhibit distinctly different properties and can be interconverted in noncoordinating organic solvents.

## Introduction

**Scheme 1:**
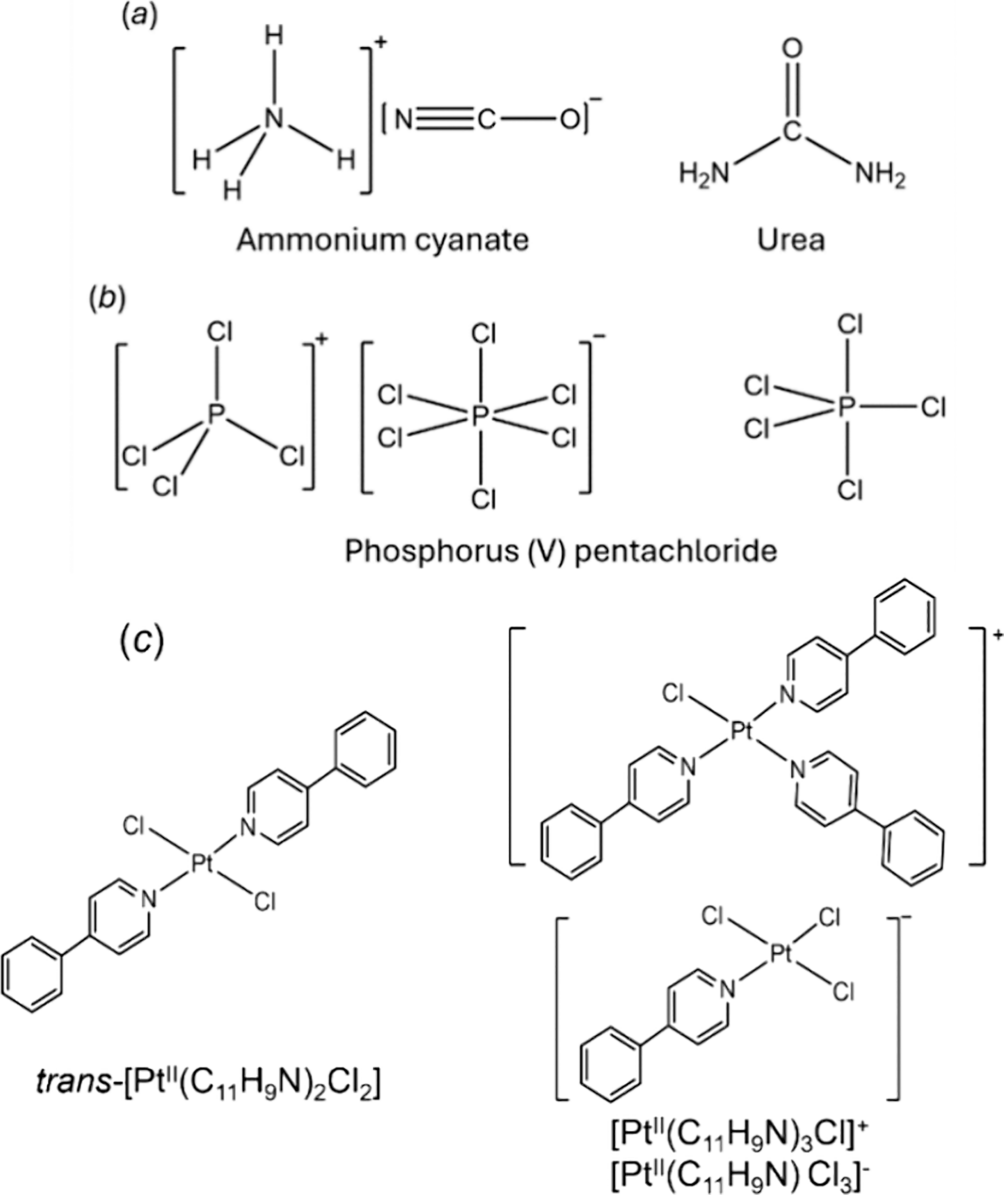
Ionic and Molecular Isomers

Evidently, the very first instance of ionic vs molecular isomers was encountered almost 200 years ago when Friedrich Wöhler synthesized urea,^1^ by slow evaporation of a water solution of ammonium cyanate (Scheme 1a). This textbook transformation is often cited as the birth of modern organic chemistry.^2^

In classification of isomers in organic chemistry, this couple is regarded as a part of structural/constitutional isomers,^3^ in which two or more organic compounds have the same formula but different structures. In the field of inorganic chemistry, a similar type of isomerism was suggested back in 1940 by Powell et al.^4^ upon confirming the solid-state structure of phosphorus(V) pentachloride as ionic {[PCl_4_]^+^[PCl_6_]^−^}, while molecular PCl_5_ exists in the gas phase and liquid state (Scheme 1b). This phenomenon was later labeled by some as “ionic isomerism”,^5,6^ with a few following studies on analogous phosphorus(V) compounds.^7,8^ For mononuclear metal complexes, two structurally characterized isomers, molecular *trans*-PtCl_2_(4-phenylpyridine)_2_ and ionic [PtCl(C_11_H_9_N)_3_]^+^[PtCl_3_(C_11_H_9_N)]^−^ (Scheme 1c), have been reported in 2011 by Ha in separate publications.^9,10^

Several types of structural isomerism are conventionally listed for metal coordination complexes such as ionization isomerism,^11^ solvate isomerism,^12^ linkage isomerism^13^ coordination isomerism,^14^ ligand isomerism,^15^ and geometric isomerism.^15^ However, none of the common classifications point out ionic/molecular isomers. Even the definition of an inorganic structural isomer often refers to a single metal center: “a difference in what ligands are bonded to the central atom or how the individual ligands are bonded to the central atom”. This definition takes the cases of isomerism in polynuclear/heterometallic assemblies somewhat out of consideration.

**Scheme 2:**
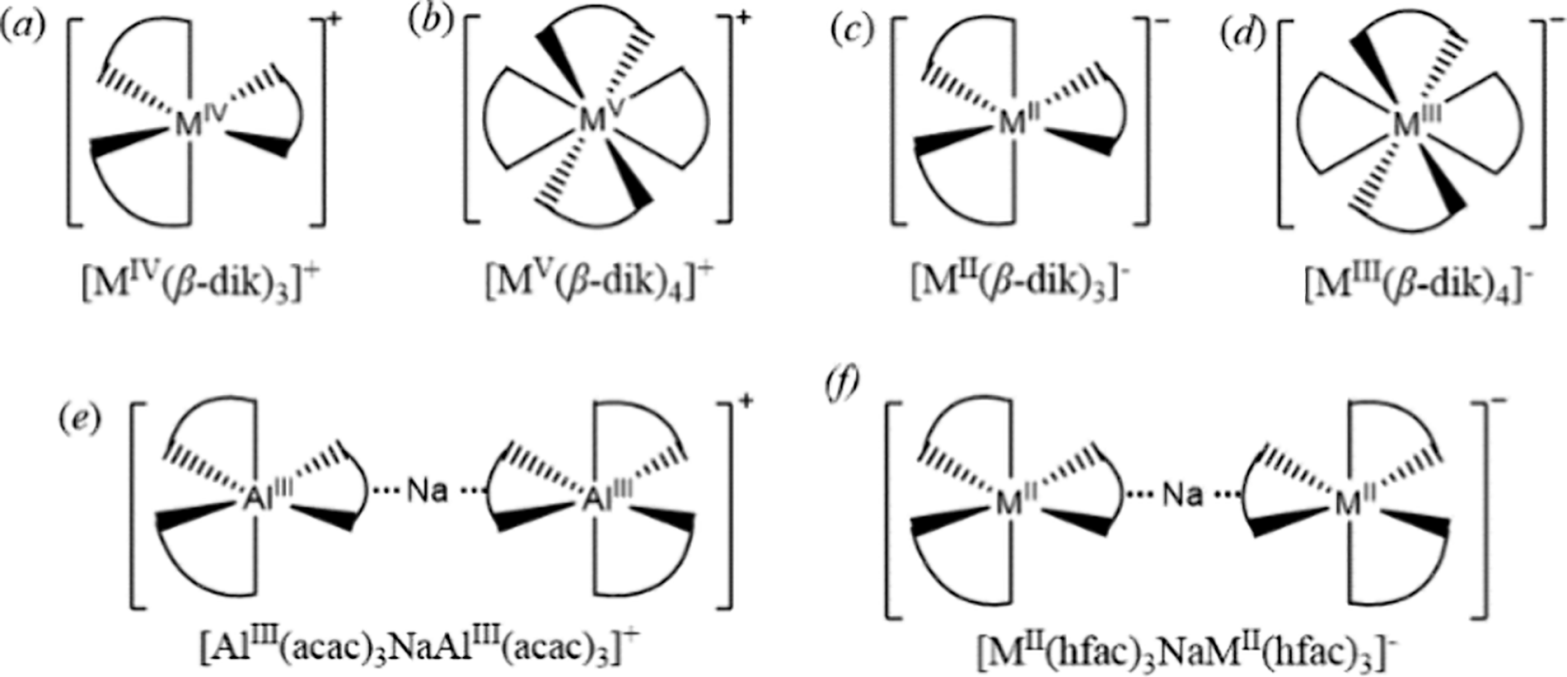
Ionic Diketonate Complexes

Metal β-diketonates are known to exhibit both molecular and ionic structures. The majority of diketonate complexes are molecular compounds that are highly volatile. At the same time, a number of metal β-diketonate fragments in crystal structures were found to be ionic, both cationic [M^IV^(β-dik)_3_]^+^ (M^IV^ = Ti, V, Ge)^16−18^ and [Ta^V^(β-dik)_4_]^+^^19^ and anionic [M^II^(β-dik)_3_]^−^ (M^II^ = Mn, Fe, Co, Ni, Cu, Zn)^20−23^ and [M^III^(β-dik)_4_]^−^ (M^III^ = La, Gd)^24,25^ (Scheme 2a–d).

We have previously reported^21^ a new class of ionic diketonates, in which both cation [Sn^IV^(thd)_3_]^+^ (Scheme 2a) and anion [M^II^(hfac)_3_]^−^ (Scheme 2c; M^II^ = Mn, Fe, and Co) are represented by metal diketonate fragments that are free of exogenous ligands or solvent molecules and exist as separate moieties. However, in these compounds, both ions are mononuclear and coordinatively saturated, making it hard to envisage the corresponding molecular isomer [SnM(thd)_3_(hfac)_3_] (thd = 2,2,6,6-tetramethyl-3,5-heptanedionate).^21^ That is the major reason, in our opinion, why having both molecular and ionic isomers is a tall order. It would require coordinatively unsaturated multimetallic/heterometallic ions (simply put, entities that can be broken into parts by coordinating solvents) to construct a molecular isomer (which will likely appear as a coordinatively unsaturated assembly itself) from those ions, but all currently known ionic diketonate fragments are mononuclear entities.

The inspiration for this investigation came from our synthesis of ionic diketonate [Al^III^(acac)_3_-Na-Al^III^(acac)_3_]^+^[Mn^II^(hfac)_3_]^−^ that contains the first trinuclear and heterometallic coordinatively unsaturated cation (Scheme 2e). The corresponding molecular compound, [Al^III^(acac)_3_-Na-Mn^II^(hfac)_3_], has also been isolated, though it is not an isomer of the above ionic assembly (synthesis and crystallographic investigation of [Al–Na–Mn] compounds can be found in the Supporting Information, pages S23–S28). Again, the problem is that the anion is a mononuclear coordinatively saturated fragment, which can hardly be combined with the complex cation. Nevertheless, this work reveals the first example of a complex coordinatively unsaturated cation. Also, we were wondering if a coordinatively unsaturated counteranion [M^II^(hfac)_3_-Na-M^II^(hfac)_3_]^−^ (Scheme 2f) can also be constructed.

In the context of this work, we are not considering zwitterionic (ionic within a molecular structure) vs molecular isomer cases, which are numerous both in the realms of organic^26,27^ and inorganic^28^ chemistry, including several instances among metal diketonates and related primarily chelating ligands.^29^ Some of those zwitterionic homo- and heterometallic diketonates do have their “neutral” molecular counterparts albeit with different metals and/or different ligands: {[Mn^II^L_3_]^−^-Mn^2+^-[Mn^II^L_3_]^−^} vs {Ni^II^L_2_-Ni^II^L_2_-Ni^II^L_2_}^30^ (L = hfac) and {[Li]^+^[Co^II^L_3_]^−^}_∞_ vs {[LiL′][Co^II^L′_2_]}_2_ (L = acac, L′ = tbaoac = *tert*-butyl acetoacetato).^31^

Herein, we report the first, to the best of our knowledge, heterometallic molecular and ionic isomers of empirical composition [NaCrFe(acac)_3_(hfac)_3_]. Molecular [Cr^III^(acac)_3_-Na-Fe^II^(hfac)_3_] (**1m**) and ionic {[Cr^III^(acac)_3_-Na-Cr^III^(acac)_3_]^+^[Fe^II^(hfac)_3_-Na-Fe^II^(hfac)_3_]^−^} (**1i**) isomers have been isolated in pure forms. Specific locations of the Cr and Fe ions as well as their oxidation states in both isomeric structures have been unambiguously confirmed by an advanced synchrotron resonant diffraction technique and synchrotron X-ray fluorescence spectroscopy, respectively. The properties of the two isomers and the transformations between them have been studied.

## Results and Discussion

### Synthesis and Properties

**Figure 1:**
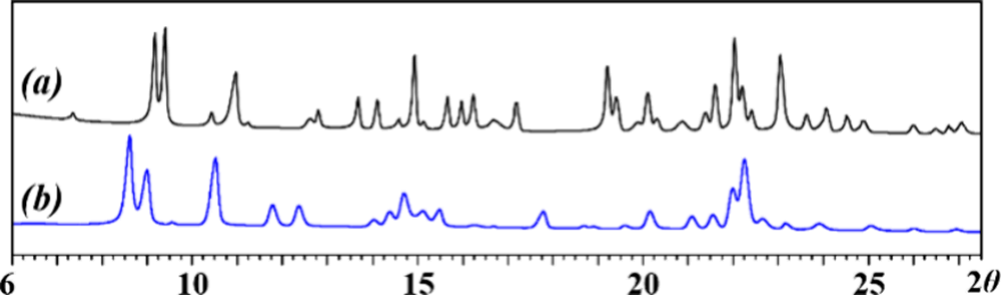
X-ray powder diffraction patterns of (a) **1m** and (b) **1i** isomers.

Two isomers with formula [NaCrFe(acac)_3_(hfac)_3_] (**1m** and **1i**) that display distinctly different X-ray powder patterns (Figure 1) have been isolated. The ICP-OES analysis of bulk materials confirmed the Na:Cr:Fe ratio of 1:1:1 for both products (see the Supporting Information, page S4). The first isomer (**1m**) was prepared through both solid-state and solution synthesis routes with high yields using the following stoichiometric reactions:
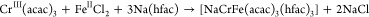
1
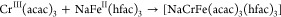
2

Reaction 1 employs commercially available Cr^III^ and Fe^II^ starting reagents. In the solid-state approach, a stoichiometric mixture of three reagents was heated to 90 °C in an evacuated sealed ampule placed in a furnace with ca. 10 °C gradient. The purple crystalline product **1m** was found to be slowly deposited in the cold section of the container, while nonvolatile NaCl remained in the hot zone. The yield is ca. 90% after a two-week sublimation. The synthesis of complex **1m** can be efficiently scaled up by performing either reaction 1 or 2 in solution. Despite the need to synthesize [NaFe(hfac)_3_] starting reagent,^32^ the reaction 2 features a much faster conversion, yielding **1m** as a sole product in a dry, oxygen-free CH_2_Cl_2_ after about 6 h of stirring at room temperature as a violet precipitate. The bulk powder of **1m** was collected upon filtration and drying the solid residue at 50 °C under vacuum overnight with the yield of ca. 96%. The reaction can also be performed in other noncoordinating haloalkanes such as CHCl_3_, C_2_H_4_Cl_2_, and C_2_H_2_Cl_4_.

The isomer **1m** has been additionally obtained by the reaction 3 that utilizes Cr^II^ and Fe^III^ as starting reagents in a 1:1 ratio, as well as by using chromium hexafluoroacetylacetonate instead of acetylacetonate (4). Both procedures have been carried out in dry and oxygen-free dichloromethane as described above, though reaction 3 can also be performed in the solid state at 90 °C in an evacuated sealed ampule.
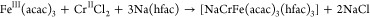
3
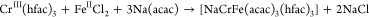
4

The second isomer (**1i**) was prepared by running the reaction 2 in anhydrous, oxygen-free hexanes at room temperature for 24 h. The isolated purple precipitate was shown to contain both **1m** and **1i** by powder X-ray diffraction analysis (Supporting Information, Figure S1). The **1i** isomer appeared as a major phase and was separated by sublimation under a static vacuum in a sealed evacuated ampule at 90 °C. While **1i** remained in the hot zone of the container, another product (**1m**) was deposited in the cold zone (80 °C). It should be noted that the mixture of **1m** and **1i** isomers can also be obtained when reactions 3 and 4 are performed in hexanes at room or elevated temperatures (see the Supporting Information, pages S4–S5).

Both isomers appear as violet solids that retain their crystallinity in the presence of oxygen in moist air for a few hours. They exhibit a poor solubility in noncoordinating, nonpolar organic solvents such as hexanes, pentane, and toluene. The **1m** isomer also has a low solubility in haloalkanes, while **1i** is soluble in these polar solvents. Coordinating solvents, such as alcohols, ketones, or THF appear to break the heterometallic assemblies. Upon solvent evaporation, the mixture of [Cr(acac)_3_] and [NaFe(hfac)_3_] has been identified in the corresponding X-ray powder diffraction spectra (Supporting Information, Figure S2). The **1m** isomer displays a very good volatility and can be quantitatively sublimed under static vacuum in a sealed, evacuated ampule starting at 70 °C, while the **1i** counterpart is not volatile under either static or dynamic (coldfinger) vacuum conditions. When the temperature is raised over 100 °C under vacuum, complex **1m** starts changing its color from violet to black, indicating decomposition. Compound **1i** displays slightly better thermal stability, tolerating temperatures of up to 130 °C under the same conditions. Phase purity of the products was confirmed by X-ray powder diffraction, and the Le Bail fit was performed to show that the experimental powder patterns of **1m** and **1i** correspond to the theoretical ones calculated from the single crystal data (Supporting Information; Figures S3 and S4, Tables S1 and S2).

### Single Crystal X-ray Structural Analysis of **1m** and **1i** Isomers

**Figure 2:**
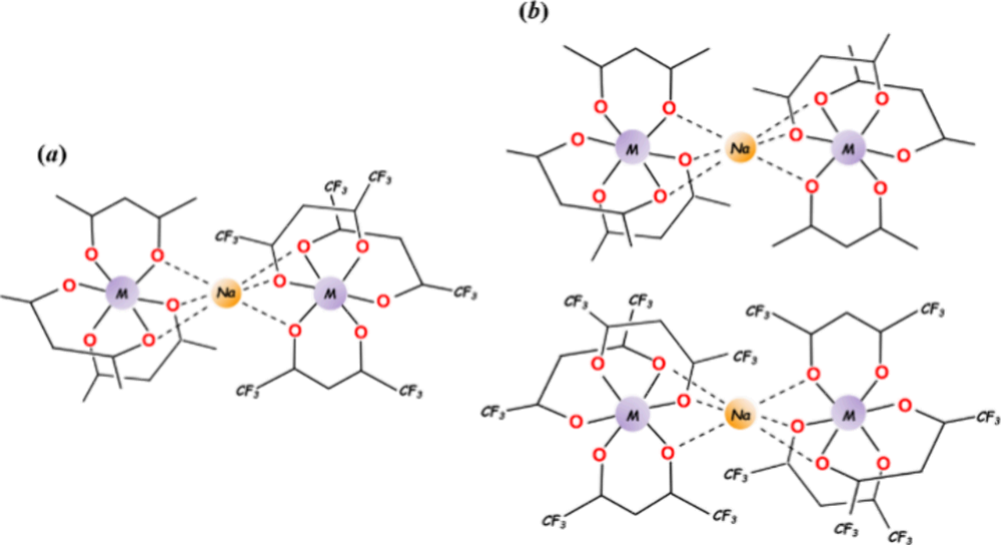
Schematic representation of (a) trinuclear unit [M(acac)_3_-Na-M(hfac)_3_] in the structure of the **1m** isomer; (b) trinuclear units [M(acac)_3_-Na-M(acac)_3_] (top) and [M(hfac)_3_-Na-M(hfac)_3_] (bottom) in the structure of the **1i** isomer.

In-house X-ray analysis of **1m** and **1i** single crystals clearly confirmed that these two complexes with the Na:Cr:Fe = 1:1:1 ratio are indeed isomers of the composition [NaCrFe(acac)_3_(hfac)_3_] as they also possess the same 1:1 ratio of acac-to-hfac ligands. Complex **1m** consists of trinuclear molecules [M(acac)_3_-Na-M(hfac)_3_] (Figure 2a) that crystallize in the centrosymmetric triclinic system and features a pair of Δ/∧ and ∧/Δ enantiomers in the unit cell. The structure of the **1i** isomer is quite different: it contains two trinuclear units [M(acac)_3_-Na-M(acac)_3_] (Figure 2b, top) and [M(hfac)_3_-Na-M(hfac)_3_] (Figure 2b, bottom). While the overall composition is the same as that in the **1m** isomer, the acac and hfac ligands are redistributed between the trinuclear units. Each unit has an inversion center at the Na position making both entities in **1i** as *meso* (Δ/∧). All trinuclear assemblies in **1m** and **1i** feature a “naked” Na ion sandwiched between two *tris*-chelated [M(acac)_3_] and/or [M(hfac)_3_] octahedral groups that provide three of their oxygen atoms each for bridging the Na centers.

Chromium and iron have close atomic numbers, masses, and ionic (+2/+3) radii (C.N. = 6, high spin in diketonate complexes).^33,34^ Therefore, the use of single crystal X-ray diffraction data for heterometallic Cr–Fe compounds to determine the exact positions of metal ions is somewhat problematic. Investigation of the **1m** isomer using in-house X-ray data resulted in the best convergence for the [Cr(acac)_3_-Na-Fe(hfac)_3_] arrangement with acac groups attached to the Cr ion and hfac ligands chelating the Fe ion (Supporting Information, Table S4). Refining the structure of **1m** as [Fe(acac)_3_-Na-Cr(hfac)_3_] leads to slightly inferior results, while the parameters for the mixed-occupancy (Cr_0.5_/Fe_0.5_ in each position) setting are close to the former model. Similarly, the refinement of **1i** structure gives the best results for the [Cr(acac)_3_-Na-Cr(acac)_3_] and [Fe(hfac)_3_-Na-Fe(hfac)_3_] arrangement, where each trinuclear unit is hetero*bi*metallic with Cr and Fe being chelated by acac and hfac ligands, respectively, similar to that in the **1m** isomer. Switching the positions of Cr and Fe results in somewhat poorer metrics (Supporting Information, Table S5), while the refinement parameters are quite comparable for the hetero*tri*metallic arrangement of [Cr(acac)_3_-Na-Fe(acac)_3_] and [Cr(hfac)_3_-Na-Fe(hfac)_3_] in both parts.

Preliminary assignment of metal positions and oxidation states in the **1m** and **1i** isomers has been attempted by comparison of the M–O bond distances (Table 1) in octahedral *tris*-chelated [M(β-dik)_3_] units. One should take into account that both **1m** and **1i** structures must contain a mixed-valent (+2/+3) combination of Cr and Fe ions to achieve electroneutrality. A quick check of the bond distances in **1m** and **1i** revealed very close M–O lengths in the corresponding [M(acac)_3_] and [M(hfac)_3_] units, indicating the same distribution of metals and charges. It should also be considered that the M–O distances in the [M(acac)_3_] and [M(hfac)_3_] units (1.95 vs 2.08 Å, respectively) are very different in both isomers, by more than can be simply explained by the difference in ionic radii of isovalent Cr and Fe ions. The latter points out different oxidation states in *tris*-chelated units, namely, [M^III^(acac)_3_] and [M^II^(hfac)_3_]. Such an arrangement is in line with the previous observations^35−38^ that the ligands with electron-donating groups (acac in this case) prefer to chelate electron-poor M^III^ ions, while those with electron-withdrawing substituents (hfac) tend to coordinate relatively electron-rich M^II^ ions.

**Table 1:** Comparison of the Averaged M–O Bond Distances in **1m** and **1i** Isomers with Those in the Corresponding [M(β-dik)_3_] (M = Cr or Fe, β-dik = acac or hfac) Units

[M(β-dik)_3_] units	[ref]	M–O_hfac_ (Å)	M–O_acac_ (Å)
**1m**	this work	2.076(2)	1.953(2)
**1i**	this work	2.077(2)	1.954(2)
[Fe^III^(acac)_3_]	(39)		1.992(2)
[Fe^III^(hfac)_3_]	(40)	1.995(2)	
[Cr^III^(acac)_3_]	(41)		1.944(2)
[Cr^III^(hfac)_3_]	(42)	1.957(2)	
[Fe^II^(acac)_3_]^−^	(43)		2.070(3)
[Fe^II^(hfac)_3_]^−^	(32)	2.082(2)	

**Figure 3:**
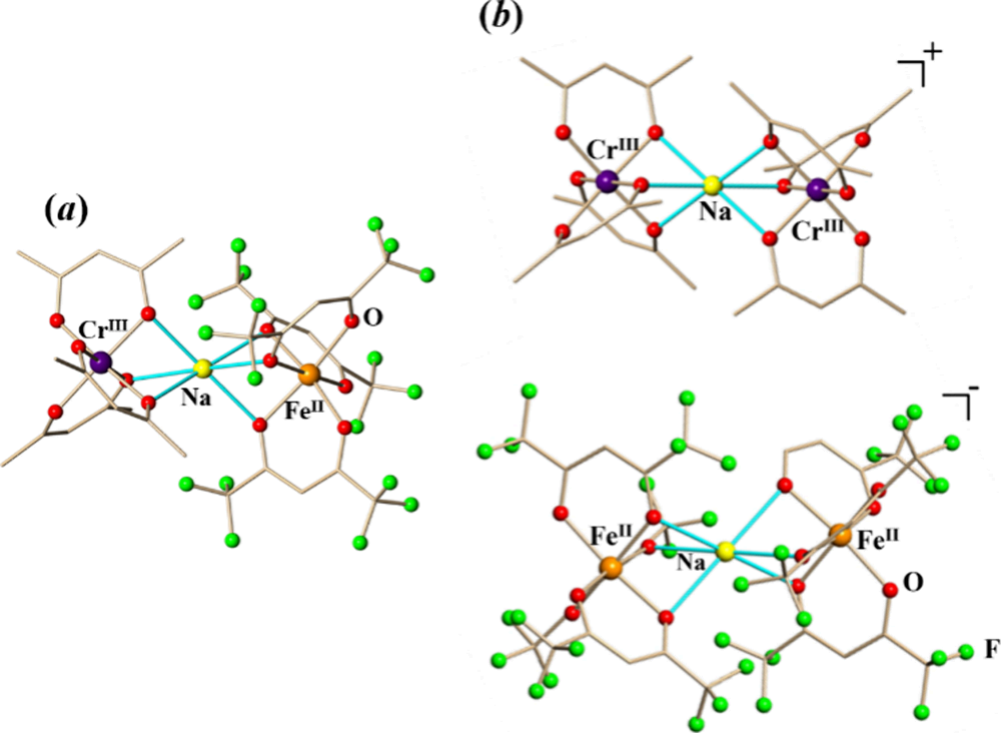
Trinuclear units in the solid-state structures of (a) **1m** and (b) **1i**. The bridging Na–O bonds are marked in blue. All hydrogen atoms have been omitted for clarity. Full view of both structures with thermal ellipsoids and the full list of bond distances and angles are included in the Supporting Information, Figures S5 and S6 and Tables S6 and S7.

Analysis of the M–O distances in the **1m** isomer (Table 1) suggests that the [M(hfac)_3_] unit is very different from both trivalent [Fe^III^(hfac)_3_] and [Cr^III^(hfac)_3_], while it is very close to the *tris*-chelated divalent [Fe^II^(hfac)_3_]^−^ anionic fragment in the structure of [NaFe^II^(hfac)_3_]. On the other hand, the [M(acac)_3_] fragment in **1m** is closer to trivalent [Cr^III^(acac)_3_] rather than to [Fe^III^(acac)_3_], while very different from divalent [Fe^II^(acac)_3_]^−^ one. These considerations support the assignment of **1m** as [Cr^III^(acac)_3_-Na-Fe^II^(hfac)_3_] (Figure 3a). Similarly, in the structure of **1i**, both [M(hfac)_3_] units are very close to divalent [Fe^II^(hfac)_3_]^−^, effectively making the corresponding trinuclear unit [Fe^II^(hfac)_3_-Na-Fe^II^(hfac)_3_]^−^ a monoanion. Conversely, both [M(acac)_3_] fragments are similar to those of trivalent [Cr^III^(acac)_3_], rendering the second trinuclear unit as a [Cr^III^(acac)_3_-Na-Cr^III^(acac)_3_]^+^ cation and thus defining the **1i** isomer as an ionic compound (Figure 3b).

As we tentatively defined two isomers as molecular [Cr^III^(acac)_3_-Na-Fe^II^(hfac)_3_] (**1m**) and ionic [Cr^III^(acac)_3_-Na-Cr^II^(acac)_3_]^+^[Fe^II^(hfac)_3_-Na-Fe^II^(hfac)_3_]^−^ (**1i**), stronger evidence is certainly required for an unambiguous assignment of transition metal positions (especially probing mixed-occupancy) and oxidation states of Cr and Fe ions.

### Unambiguous Assignment of the Metal Positions and Oxidation States of Cr and Fe Ions in **1m** and **1i** Isomers

**Figure 4:**
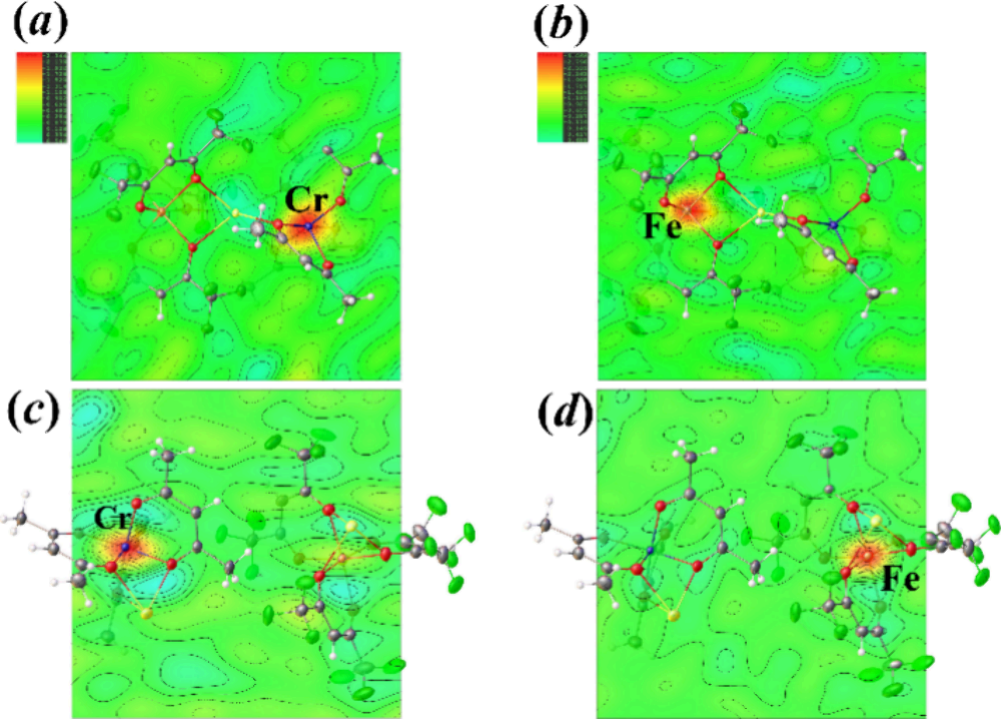
Difference Fourier electron density maps at absorption K-edges of (a) Cr in **1m**, (b) Fe in **1m**, (c) Cr in **1i**, and (d) Fe in **1i**. Only the maps for crystallographically independent parts are shown for the **1i** isomer.

To precisely determine each metal site occupancy factor, synchrotron X-ray resonant single crystal diffraction investigations were carried out for both isomers **1m** and **1i** utilizing the advantages of the sensitive *K*-edge absorption at the characteristic wavelengths. This method has been demonstrated^37,38,44^ to successfully distinguish Periodic Table neighbors based on significant differences in the anomalous dispersion factors of the elements around their absorption edges. A total of five data sets at different wavelengths (two near the Cr *K*-edge, two near the Fe *K*-edge, and one away from the above absorption edges, i.e., 30 keV) were collected by using a synchrotron radiation source. The structural models derived from the 30 keV data were refined against those data sets near both *K*-edges to analyze the composition of both transition metal positions in the [M(acac)_3_] and [M(hfac)_3_] units. Analysis of the anomalous difference Fourier electron density maps provides visual pictures of the metal site occupancy patterns. Data sets measured at the wavelengths near the *K*-edges (Figure 4) show deep electron density holes for the respective crystallographically independent metal positions thus revealing the presence of only Cr in the [M(acac)_3_] units (Figure 4a,c) and only Fe in the [M(hfac)_3_] fragments (Figure 4b,d) of both isomeric structures. The corresponding site occupancy factor refinements give the ratios of Cr:Fe as 1.02(1):1.02(1) and 1.033(11):1.009(12) in **1m** and **1i**, respectively (see the Supporting Information, pages S10–S11 for detailed experimental procedures).

**Figure 5:**
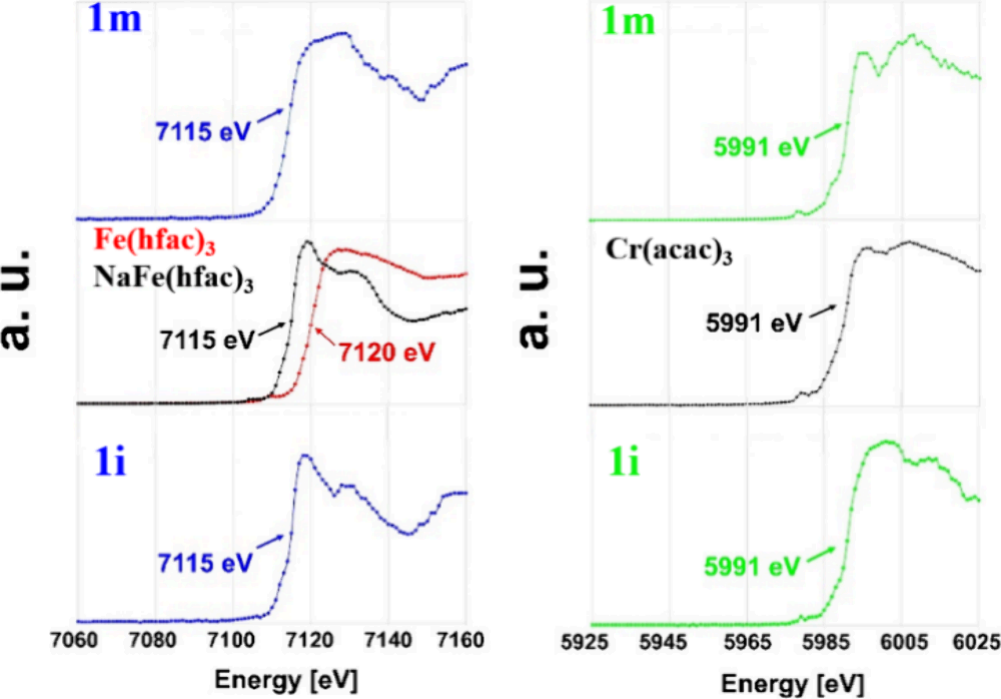
X-ray fluorescence scans collected in steps of 1 eV at 100 K around: the Fe *K*-edge for the crystalline powder of [NaFe^II^(hfac)_3_] (middle-left, black) and [Fe^III^(hfac)_3_] (red) compared with the anomalous scattering factor *f* plots of the Fe position in the structures of **1m** (top-left) and **1i** (bottom-left); and the Cr *K*-edge for the crystalline powder of [Cr^III^(acac)_3_] (middle-right) compared with the anomalous scattering factor *f* plots of the Cr position in the structures of **1m** (top-right) and **1i** (bottom-right).

After Cr and Fe have been precisely located with full occupancy in the [Cr(acac)_3_] and [Fe(hfac)_3_] units, X-ray fluorescence spectroscopy (see the Supporting Information, pages S10–S11 for detailed experimental procedures) was carried out for the **1m** and **1i** structures to reveal the oxidation states of the Cr and Fe ions (Figure 5). This element-specific method with high chemical sensitivity allows distinguishing different oxidation states of the probed elements. The fluorescence spectrum globally shifts toward higher energy with the increase of the element formal oxidation state.^45^ By comparison of the element *K-*edge in the structure under investigation with *K-*edges in standards of the same element with similar coordination environments, the formal oxidation state can be determined. The X-ray fluorescence spectra of both **1m** and **1i**, as well as Cr and Fe standards with different oxidation states, were recorded using a synchrotron radiation source. The *K-*edge values were extracted from the first-order derivative of each spectrum. It was found that both isomers exhibit the same Cr *K-*edge energy (5991 eV) as in its trivalent standard, [Cr^III^(acac)_3_], as well as the same Fe *K-*edge energy (7115 eV) as in its divalent standard, [NaFe^II^(hfac)_3_], clearly confirming the presence of Cr^III^ and Fe^II^ in heterometallic assemblies **1m** and **1i**.

The combination of trivalent Cr and divalent Fe in heterometallic isomeric structures **1m** and **1i** corresponds to their redox potentials^46^ as well as to the analysis of heterometallic Cr–Fe structures in the CCDC database.^47^ About 60 of the Cr^III^–Fe^II^ mixed-valent compounds that include both molecular and ionic structures were identified with no indication of Cr^II^–Fe^III^ combination present. Correct assignment of the Cr and Fe positions and oxidation states in the **1m** and **1i** isomers allows us to further analyze the synthetic procedures. Apparently, reaction 3 represents a redox process Cr^2+^ + Fe^3+^ = Cr^3+^ + Fe^2+^, while both reactions 3 and 4 indicate ligand exchange with an electron-donating acac group chelating the electron-poor Cr^III^ ions and electron-withdrawing hfac coordinating Fe^II^.

### Characterization of **1m** and **1i** Isomers

Mössbauer spectroscopy allows one to sensitively examine the changes in the energy levels of an atomic nucleus in response to its oxidation states and environment. In this work, the Mössbauer spectra for isomers **1m** and **1i** were collected at low temperature (4.2 K) to confirm the oxidation states for the Fe ions in each structure.

**Figure 6:**
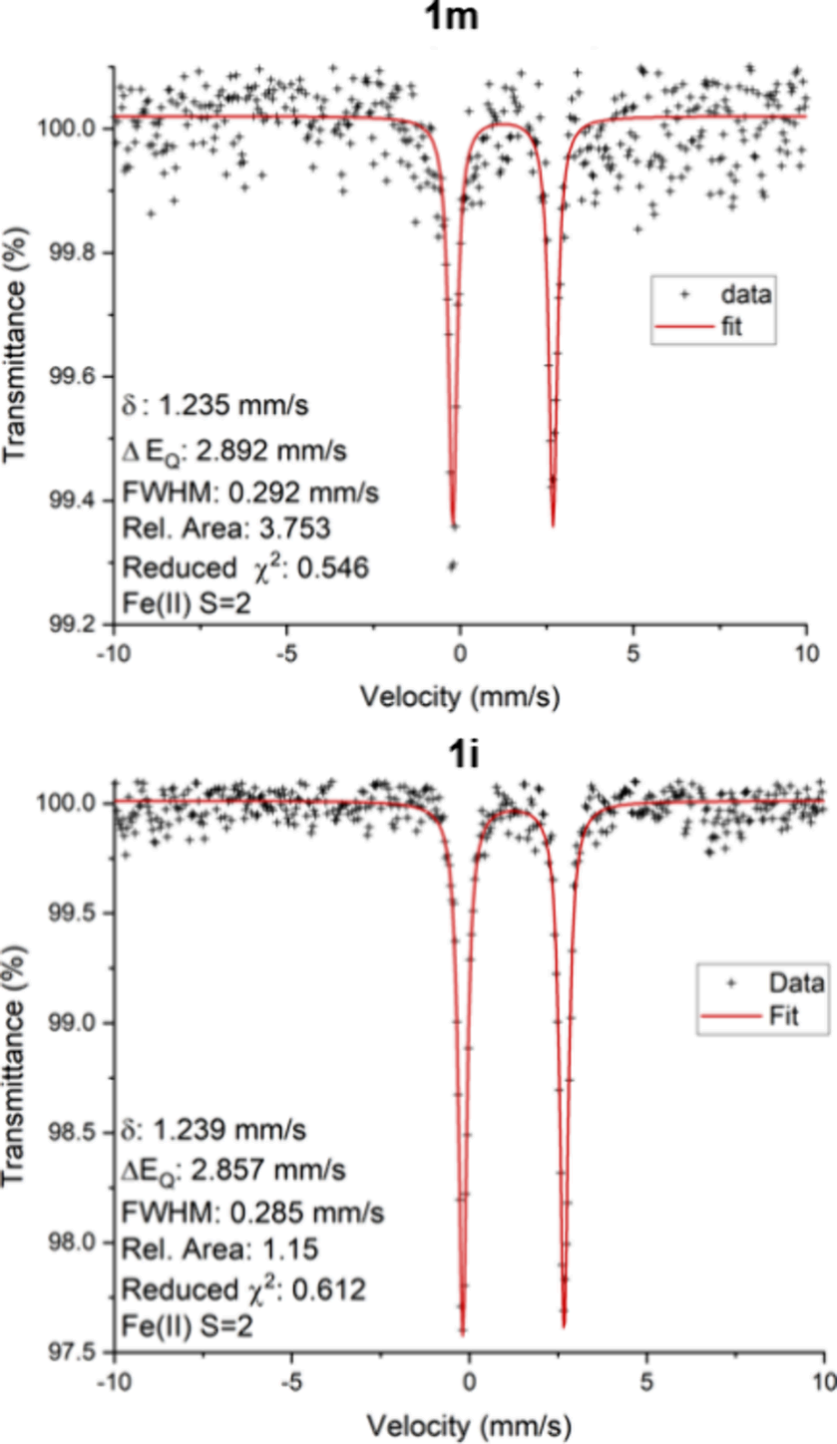
Low-temperature Mössbauer spectra of isomers **1m** (top) and **1i** (bottom) with parameters from the fit. The data are displayed as black plus signs, and the fit to the data is shown as red lines.

In the spectra of two isomers (Figure 6), the values of the isomer shift δ for **1m** (up) and **1i** (bottom) are practically identical, 1.24 mm·s^–1^, as are the quadrupole splitting Δ*E*_Q_ values ∼2.88 mm s^–1^. These parameters are uniquely indicative of high-spin Fe^II^ ions.^48^ The similarities reflect the similar chemical environments of the Fe^II^ ions in the two isomers. The Mössbauer parameters compare well with those of related compounds. Interestingly, Fe(II) acetylacetonate was reported to have no Mössbauer signal,^49^ though we reported a Mössbauer spectrum of the [Fe^II^(ptac)_3_]^−^ unit (δ = 1.15 mm s^–1^, Δ*E*_Q_ = 2.31 mm s^–1^)^36^ that agrees well with the spectral features of **1m** and **1i**. Fe^II^ oxalate, in which an Fe^II^ is also in an all O-donor environment, also has reported Mössbauer parameters (δ = 1.17 mm s^–1^, Δ*E*_Q_ = 1.68 mm s^–1^);^50^ the smaller Δ*E*_Q_ value for the oxalate likely reflects a higher symmetry structure. We may also compare the Mössbauer parameters to those of ilmenite, FeTiO_4_, a mixed-metal ferrous mineral (δ ∼1.05 mm s^–1^, Δ*E*_Q_ ∼0.72 mm s^–1^)^51^ in which Fe^II^ is in a six-coordinate, all-O-donor environment. The smaller Δ*E*_Q_ in ilmenite indicates a more rigorously octahedral crystal site, and the lower isomer shift likely reflects a degree of charge transfer in the mineral (partial Fe^III^/Ti^III^ character). Notably, the higher isomer shifts in **1m** and **1i** indicate a lack of such a charge transfer character in the compounds reported here. All of the observations above agree with the X-ray structural analysis that high-spin divalent iron ions in the two isomers are both *tris-*chelated to the same hfac ligands. This also agrees with standard M^3+^/M^2+^ reduction potentials: *E*_0_ (Fe^3+^/Fe^2+^) = +0.77 and *E*_0_ (Cr^3+^/Cr^2+^) = −0.424.^52^

**Figure 7:**
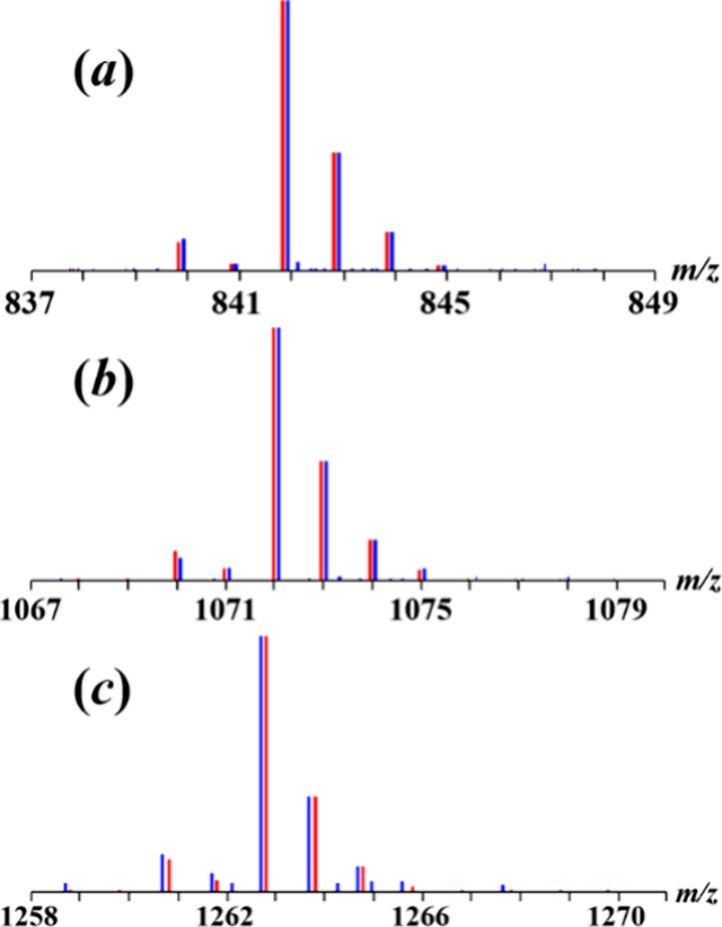
Isotope distribution patterns for (a) [M – hfac]^+^ (M = [NaCrFe(acac)_3_(hfac)_3_]) and (b) [M + Na]^+^ ions in the positive mode DART mass spectrum of **1m**; (c) [M + hfac]^−^ ion in the negative mode DART mass spectrum of **1m**. Blue and red lines represent experimental and calculated patterns, respectively.

Direct Analysis in Real Time (DART) mass spectrometry has been successfully utilized to confirm the composition of heterometallic ions through their isotope distribution patterns, as well as to analyze the oxidation states of constituent transition metals.^53^ Mass spectra of **1m** and **1i** in both positive and negative modes (Supporting Information, Figures S7–S10) are clearly different, allowing to distinguish two isomers, even in trace quantities. In the positive mode, the mass spectrum of **1m** features the heterometallic peaks [M – hfac]^+^ (M = [NaCrFe(acac)_3_(hfac)_3_], meas/calcd = 841.980/841.975, Figure 7a) and [M + Na]^+^ (meas/calcd = 1071.959/1071.953, Figure 7b). Those appear with characteristic isotope distribution patterns in good agreement with the simulated patterns, confirming the presence of hetero*tri*metallic trinuclear molecules in the gas phase. These two peaks are notably absent in the mass spectrum of the ionic isomer **1i**. Similarly, in the negative mode, the [M+hfac]^−^ peak (meas/calcd = 1262.889/1262.906, Figure 7c) is found only in the mass spectrum of molecular isomer **1m**. Importantly, all homometallic peaks in the mass spectra of **1m** and **1i** (Supporting Information, Tables S8–S11) correspond to the oxidation states of Cr and Fe as +3 and +2, respectively.

**Figure 8:**
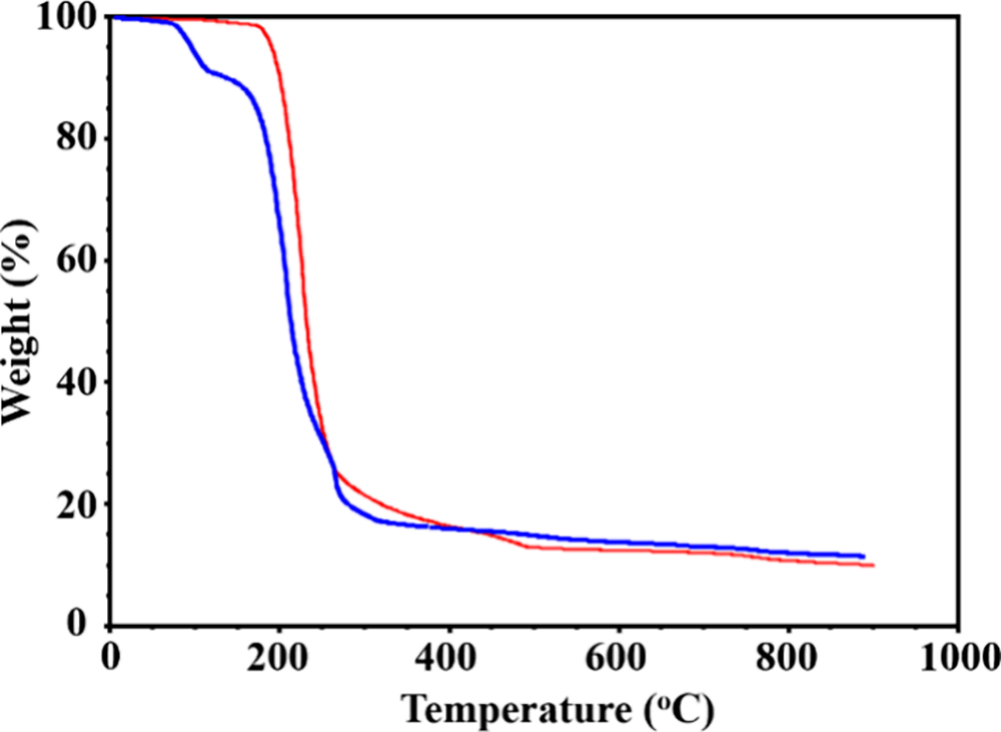
TGA plots of **1m** (blue) and **1i** (red) isomers recorded with a heating rate of 1.0 °C/min under a 25 mL/min argon protection flow.

TGA analysis (Figure 8) first revealed the differences in volatility of the **1m** and **1i** isomers (see the Supporting Information, page S3 for detailed TGA settings). Molecular isomer **1m** shows a characteristic mass loss due to sublimation starting at 75 °C before decomposition, while the ionic isomer barely lost any weight until around 150 °C. The weight loss curve is sharp for both isomers between 150 and 300 °C. The continuous weight drop at higher temperatures likely indicates the loss of Na, as it has been previously observed for some sodium-containing heterometallic precursors.^38,54^ Powder X-ray diffraction analysis of decomposition traces (Supporting Information, Figure S11) revealed the appearance of Na_0.5_Fe_*x*_Cr_1–*x*_O_2_^55,56^ oxide as a major phase upon thermolysis of **1m**, while **1i** produced a mixture of Fe_2_O_3_ and Na_2_CrO_4_^57^ under the same conditions (Supporting Information, Figure S12).

### Transformation between Molecular and Ionic Isomers

The possible conversion between two isomeric forms has been studied in solid-state, gas-phase, and solution environments. Clearly, there is no transformation between **1m** and **1i** in the solid state (crystal-to-crystal) as monitored by the X-ray powder diffraction technique upon heating up both isomers for a prolonged time under anaerobic conditions at temperatures slightly below their respective decomposition points. Similarly, no transformation has been detected in the gas phase upon checking the powder X-ray diffraction patterns of complex **1m** sublimation products at different temperatures under a static vacuum. The molecular isomer retains its structure when the sublimation temperature is kept below 90 °C, while it starts disintegrating to a mixture of [Cr^III^(acac)_3_] and an unknown phase when the heat is raised to close to 130 °C.

**Figure 9:**
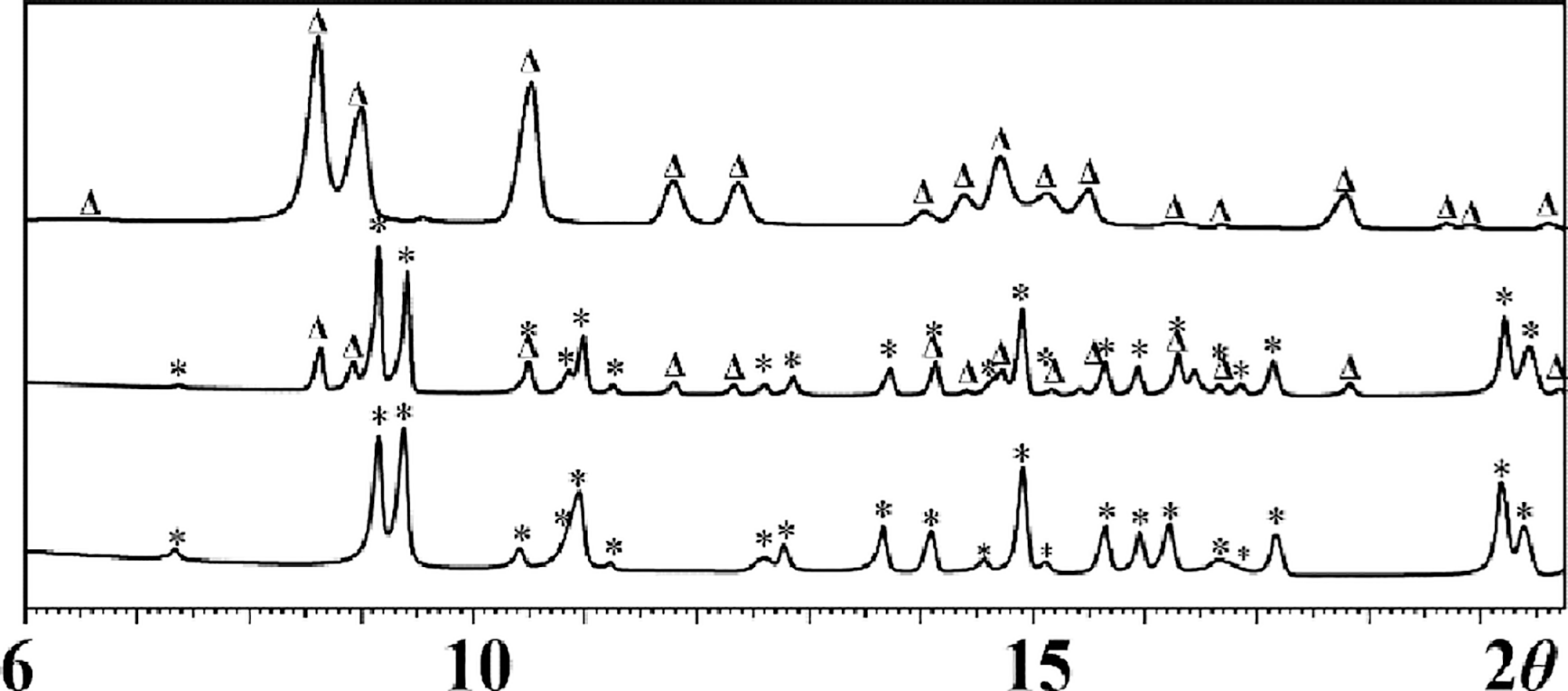
X-ray powder diffraction patterns (2θ = 6–20°) of the residues obtained upon evaporation of solvent after dissolving the **1i** isomer in dichloromethane at room temperature: right after dissolution (top);after 10 min (middle); after 2 hours (bottom). The Δ and * labels designate **1i** and **1m** theoretical peak positions, respectively.

The transformation between two isomers was observed in solutions of noncoordinating solvents only since both structures react to produce homometallic fragments in coordinating solvents as described above. The transformation from **1i** to **1m** clearly takes place in solutions of polar haloalkanes such as dichloromethane or chloroform. Dissolving **1i** in dry, deoxygenated dichloromethane initially results in a purple solution, but stirring it at room temperature generates a purple precipitate after a few minutes. X-ray powder diffraction analysis of the solid residue obtained upon solvent evaporation unambiguously confirmed the complete transformation of **1i** into **1m** after 2 h (Figure 9).

**Figure 10:**
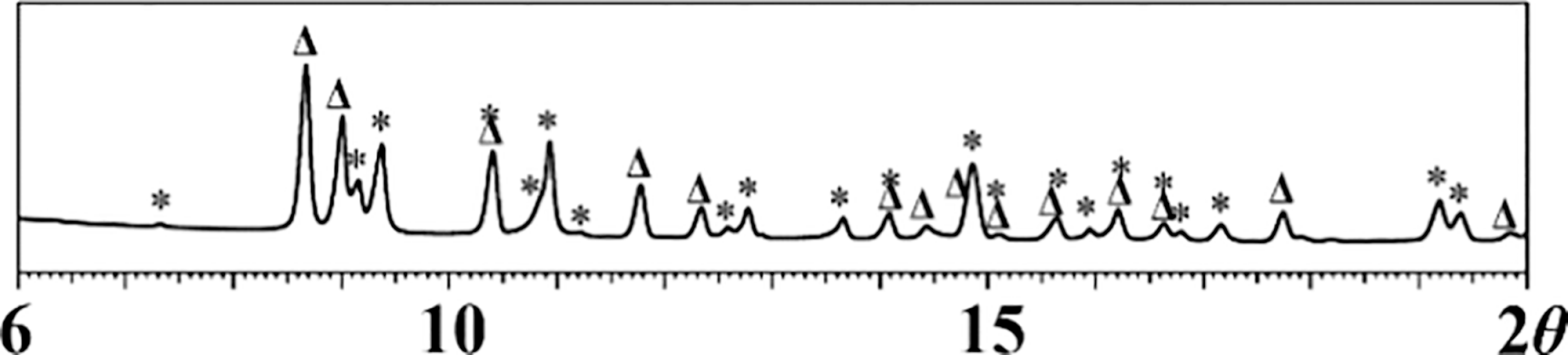
X-ray powder diffraction pattern (2θ = 6–20°) of the solid residue obtained upon evaporation of solvent after dissolving the **1m** isomer in hexanes and stirring it at 40 °C for a week. The Δ and * labels designate **1i** and **1m** theoretical peak positions, respectively.

The reverse transformation was found to occur in hexanes. Crystals of **1m** and **1i** were crystallized from the supersaturated **1m** hexanes solution in a sealed ampule under an argon atmosphere at room temperature after 3 days. We did not observe the complete transformation of **1m** to **1i** in hexanes at room temperature, even after a month. Upon stirring a saturated solution of **1m** in hexanes at 40 °C under an argon atmosphere for 1 week, a mixture of **1i** and **1m** was still detected by powder X-ray diffraction (Figure 10) after solvent evaporation.

Apparently, low solubility appears as the major factor in isolation of these isomers from solution. While we have no means to analyze if the equilibrium between **1i** and **1m** exists in solution, one particular observation is noteworthy. The DART mass spectrum of hexane solution of **1m** after 1 week does not show the ions [M – hfac]^+^ and [M + Na]^+^ (M = [NaCrFe(acac)_3_(hfac)_3_]) characteristic of molecular isomer **1m** (Figure 7a,b), indicating that the transformation could be complete. It should be noted that obtaining DART spectra of solutions still involves solvent evaporation, though it is a fast process compared to those processes of crystal growth or bulk powder isolation upon solvent removal under vacuum. Even if the complete transformation from **1m** to **1i** does take place in hexanes solution, the former isomer still appears in the X-ray powder pattern as the minor product upon solvent evaporation.

## Conclusions

We have confirmed in this study that heterometallic complexes may appear as molecular isomers and ionic isomers. It ultimately required two coordinatively unsaturated complex ions to produce a neutral coordinatively unsaturated molecule upon recombination: [A-B-A]^+^ + [C–B–C]^−^ = 2[A–B–C] (A = [Cr^III^(acac)_3_]; B = Na^+^; C = [Fe^II^(hfac)_3_]^−^). Also, while both ions are hetero*bi*metallic trinuclear entities, the neutral counterpart is a hetero*tri*metallic trinuclear molecule. We keep looking for other examples of such isomerism, wondering why the Cr–Na–Fe system appears as a unique one. While the neutral trinuclear assembly [M^III^-Na-M^II^] seems to be instantly achieved with nearly any combination of divalent and trivalent metals (both same and different, main group and transition metals), those systems are not eager to display the presence of ionic isomers.

One can expect more interesting cases of isomerism in hetero*multi*metallic polynuclear compounds that are not feasible for their homometallic or homonuclear counterparts. Another type of isomerism that one can envision in heterometallic polynuclear assemblies is a combination of different types, e.g., the structural isomerism blended with stereoisomerism,^58^ since *tris*-chelated and some of the *bis*-chelated metal complexes exhibit optical isomerism. In polynuclear complexes containing two or more chiral centers, diastereomers are possible. Attention to the preparation conditions (starting reagents, solvents, temperature, gas phase vs solid-state reactions) and thorough investigation of bulk reaction products (especially by powder X-ray diffraction and DART mass spectrometry) are the keys for revealing yet unknown types of inorganic isomers.

## Experimental Section

### General Procedures

Hexafluoroacetylacetate (Hhfac) was purchased from Sigma-Aldrich and used as received. Anhydrous iron(II) chloride (FeCl_2_), anhydrous chromium(II) chloride (CrCl_2_), sodium methoxide (NaOMe), and sodium acetylacetonate (Na(acac)), chromium(III) acetylacetonate (Cr(acac)_3_), iron(III) acetylacetonate (Fe(acac)_3_), and chromium(III) hexafluoroacetylacetonate (Cr(hfac)_3_) were purchased from Sigma-Aldrich and used as received after checking their X-ray powder diffraction patterns. Sodium hexafluoroacetylacetonate (Na(hfac)) was synthesized by previously reported procedure.^32^ The ICP-OES analyses were carried out on an ICPE-9820 plasma atomic emission spectrometer, Shimadzu. The DART-MS spectra were recorded on a JEOL AccuTof 4G LC-plus DART mass spectrometer over the mass range of *m*/*z* 50–2000 at one spectrum per second with a gas heater temperature of 300 °C. X-ray powder diffraction data were collected on a Rigaku multipurpose θ–θ X-ray SmartLab SE diffractometer (Cu *K*_α_ radiation, HyPix-400 two-dimensional advanced photon counting hybrid pixel array detector, step of 0.01° 2θ, 20 °C). Le Bail fit for powder diffraction patterns has been performed using the TOPAS version 4 software package (Bruker AXS, 2006). Thermogravimetric analysis (TGA) was carried out under 25 mL/min argon protection flow at a heating rate of 0.1–1 °C/min using a TGA 5500 (TA Instruments-Waters LLC). All Mössbauer data were collected with a See Co model W304 resonant gamma-ray 1024 channel spectrometer with a ^57^Co on Rh foil source. Data collection was conducted at 4.2K with the sample under a vacuum. Single crystal diffraction data were measured at 100(2) K on a Huber Kappa 4-Circle diffraction system with a DECTRIS PILATUS3 × 2 M (CdTe) pixel array detector using ϕ scans (synchrotron radiation at λ = 0.41328 Å) located at the Advanced Photon Source, Argonne National Laboratory.

### Synthesis

Heterometallic molecular isomer [Cr(acac)_3_-Na-Fe(hfac)_3_] (**1m**) has been obtained by using both solid-state and solution techniques. In the solution reaction, the mixture of [Cr(acac)_3_] and [NaFe(hfac)_3_] was stirred for 6 h in dry, oxygen-free dichloromethane. The yield was ca. 96%. ICP-OES (2% HNO_3_ water solution, 20 °C): Cr, 4.80% (Calcd: 4.96%); Na, 2.20% (2.19%); Fe: 5.15% (5.32%). In the solid-state method, [Cr(acac)_3_], anhydrous FeCl_2_ and Na(hfac) were ground in a glovebox under an argon atmosphere and sealed in a 10 cm long evacuated ampule. The ampule was placed in a gradient furnace at 90 °C with a temperature difference of ca. 10 °C. Purple crystals grew in the cold zone of a container after 2 weeks. The yield was ca. 90%. The molecular isomer **1m** has also been obtained by redox reaction of [Fe(acac)_3_] with CrCl_2_ and Na(hfac) performed either in dichloromethane solution at room temperature or by the solid-state reaction run at 90 °C in an ampule with a temperature gradient. In addition, the ligand-exchange reaction of [Cr(hfac)_3_] with anhydrous FeCl_2_ and Na(acac) also resulted in the **1m** isomer after stirring the mixture under an argon atmosphere for 24 h at room temperature. Heterometallic ionic isomer **1i** has been obtained by solution reaction of [Cr(acac)_3_] with [NaFe(hfac)_3_] under a dry argon atmosphere in dry, oxygen-free hexanes for 24 h at room temperature. The yield was ca. 50%. ICP-OES (2% HNO_3_ water solution, 20 °C): Cr, 4.94% (Calcd: 4.96%); Na, 2.15% (2.19%); Fe: 5.20% (5.32%). The detailed experimental conditions for different synthetic methods are summarized in the Supporting Information, pages S4–S5.

## Supporting Information Available

Experimental details, synthesis, ICP-OES data, X-ray powder diffraction patterns, crystal growth conditions, X-ray crystallographic procedures, synchrotron X-ray resonant diffraction and X-ray fluorescence spectroscopy protocols, single crystal structures with bond distances and angles, DART-MS and Mössbauer spectra, and powder diffraction patterns of thermal decomposition products; additional file of crystallographic data PDF)
