# Supplementary material for: Heterometallic Molecular and Ionic Isomers
Source: Inorg Chem. 2024 Oct 4;63(41):19499–508. doi: 10.1021/acs.inorgchem.4c03849 (PMC11483736; doi:10.1021/acs.inorgchem.4c03849)
Supplement: Supplementary file 1 — ic4c03849_si_001.pdf [file ic4c03849_si_001.pdf]

Supporting Information

Heterometallic Molecular and Ionic Isomers

Yuxuan Zhang[a], Zheng Wei[a], Haixiang Han[b], Joyce Chang[a], Samantha Stegman[c], Tieyan Chang[d], Yu-Sheng Chen[d], John F. Berry,[,c] and Evgeny V. Dikarev*[,a]*

[a] *Department of Chemistry, University at Albany, SUNY, Albany, NY 12222, United States*

[b] *School of Material Science and Engineering, Tongji University, Shanghai, 201804, China*

[c] *Department of Chemistry, University of Wisconsin-Madison, Wisconsin 53706, United State*

[d] *NSF's ChemMatCars, Center for Advanced Radiation Source, The University of Chicago, Argonne, IL 60439, United States*

*Author to whom correspondence should be addressed. E-mail : edikarev@albany.edu. Phone : (518)442-4401. Fax: (518)442-3462.

Table of Contents

1. General Procedures 3

2. Synthesis of Heterometallic Isomers **1m** and **1i** 4

3. X-Ray Powder Diffraction Patterns of **1m** and **1i** Isomers 6

4. Single Crystal Growth 9

5. X-Ray Crystallographic Procedures 10

6. X-Ray Single Crystal Refinement of Different Assignments of Fe and Cr Site Occupancy in **1m** and **1i** Isomers 13

7. Solid State Structures of Molecular and Ionic Isomers 14

8. Direct Analysis in Real Time (DART) Mass Spectra of **1m** and **1i** Isomers 18

9. Thermal Decomposition Patterns of **1m** and **1i** Isomers 22

10. Synthesis, Crystallization, and Structural Analysis of Molecular $[Al(acac)_3NaMn(hfac)_3]$ and Ionic $\{[Al(acac)_3NaAl(acac)_3]^+[Mn(hfac)_3]^-\}$ Compounds. 23

11. References 29

1. General Procedures

Hexafluoroacetylacetate (Hhfac) was purchased from Sigma-Aldrich and used as received. Anhydrous iron(II) chloride ($FeCl_2$), anhydrous manganese(II) chloride ($MnCl_2$), anhydrous chromium(II) chloride ($CrCl_2$), sodium methoxide (NaOMe), sodium acetylacetonate (Na(acac)), chromium(III) acetylacetonate ($Cr(acac)_3$), iron(III) acetylacetonate ($Fe(acac)_3$), chromium(III) hexafluoroacetylacetonate ($Cr(hfac)_3$)) and aluminum acetylacetonate ($Al(acac)_3$) were purchased from Sigma-Aldrich and used as received after checking their X-ray powder diffraction patterns. Sodium hexafluoroacetylacetonate (Na(hfac)) was synthesized by previously reported procedure.[1] The ICP-OES analyses were carried out on ICPE-9820 plasma atomic emission spectrometer, Shimadzu. The DART-MS spectra were recorded on a JEOL AccuTof 4G LC-plus DART mass spectrometer over the mass range of *m/z* 50–2000 at one spectrum per second with a gas heater temperature of 300 °C. X-ray powder diffraction data were collected on a Rigaku multipurpose θ-θ X-ray SmartLab SE diffractometer (Cu Kα radiation, HyPix-400 two-dimensional advanced photon counting hybrid pixel array detector, step of 0.01° 2θ, 20 °C). Le Bail fit for powder diffraction patterns has been performed using TOPAS version 4 software package (Bruker AXS, 2006). Thermogravimetric (TGA) measurements were carried out under 25 mL/min argon protection flow at a heating rate of 0.1-1 °C/min using a TGA 5500 (TA Instruments-Waters LLC). All Mössbauer data were collected with a See Co model W304 resonant gamma-ray 1024 channel spectrometer with a ^{57}Co on Rh foil source. The velocity range used was +/- 10 mm/s. Data collected was conducted at 4.2K with the sample under vacuum. Mössbauer data were fitting using the WMOSS4F[2] software package. The theoretical model used to treat the data to fit a quadrupole doublet to the experimental data. The fitting method used for each fit was an adaptive non-linear least-square algorithm developed by Dennis et al[3] available within the WMOSSF software. The parameters fit for each quadrupole doublet were their isomer shift (∂), quadrupole splitting (ΔEq), linewidths (i.e. FWHM) and the relative areas (peak integrations).

2. Synthesis of Heterometallic Isomers 1m and 1i

[Cr(acac)$_3$NaFe(hfac)$_3$] (1m)

1) Solution method: A 50 mL Schlenk flask was charged with a mixture of [Cr(acac)$_3$] (0.050 g, 0.143 mmol) and [NaFe(hfac)$_3$][1] (0.100 g, 0.143 mmol) under dry argon atmosphere, and 15 mL of dry, oxygen-free dichloromethane was added. Purple precipitate started to appear immediately, and the reaction was stirred under argon atmosphere at room temperature for 6 hours. The solvent was then removed under vacuum, and the purple solid residue (**1m**) was dried under vacuum at 50-60 ºC sand bath overnight. The yield was *ca*. 0.144 g (96%). The purity of the crystalline product was confirmed by powder X-ray diffraction analysis (Figure S3 and Table S1). ICP-OES (2% HNO_3 water solution, 20 ºC): Cr, 4.80% (Calcd: 4.96%); Na, 2.20% (2.19%); Fe: 5.15% (5.32%).

2) Solid-state method: [Cr(acac)$_3$] (0.041 g, 0.118 mmol), anhydrous $FeCl_2$ (0.015 g, 0.118 mmol) and Na(hfac) (0.081 g, 0.354 mmol) were grinded in glovebox under argon atmosphere and sealed in 10 cm long evacuated ampule. The ampule was placed in a gradient furnace at 90 ºC with a temperature difference of *ca.* 10 ºC. Purple crystals grow in the cold zone of a container after 24 hours. After 2-week sublimation, purple crystals of **1m** were deposited in the cold zone of the ampule, while the white non-volatile NaCl remained at the hot zone. The yield was *ca.* 0.111 g (90%).

3) Redox reaction, solution method: A 50 mL Schlenk flask was charged with a mixture of [Fe(acac)$_3$] (0.057g, 0.161 mmol), anhydrous $CrCl_2$ (0.020 g, 0.162 mmol) and Na(hfac) (0.111 g, 0.485 mmol) under dry argon atmosphere, and 20 mL of dry, oxygen-free dichloromethane was added. The reaction was stirred at room temperature under the argon atmosphere. The solution color turned from red to purple after about one hour reaction accompanied by the appearance of purple precipitate. After 24 hours of stirring, the purple solution was filtered off, and 2 mL of it was sealed in a 10 cm long ampule under argon atmosphere that was placed into a –10 ºC freezer. After 3 days, block-shaped, purple crystals of **1m** were crystallized on the walls of the ampule.

3-1) Redox reaction, solid-state method: [Fe(acac)$_3$] (0.035 g, 0.099 mmol), anhydrous $CrCl_2$ (0.012 g, 0.098 mmol) and Na(hfac) (0.069 g, 0.301 mmol) were grinded in glovebox under argon atmosphere and sealed in 10 cm long evacuated ampule. The ampule was placed in a gradient furnace at 90 ºC with a temperature difference of *ca.* 10 ºC. The reaction residue color at the hot zone of the ampule gradually turned from red to dark purple during 24 hours, while purple crystals of **1m** started to grow in the cold section of container in the next 72 hours.

4) Ligand-exchange reaction: A 50 mL Schlenk flask was charged with a mixture of [Cr(hfac)$_3$] (0.079g, 0.118 mmol), anhydrous $FeCl_2$ (0.015 g, 0.118 mmol) and Na(acac) (0.043 g, 0.353 mmol) under dry argon atmosphere, and 20 mL of dry, oxygen-free dichloromethane was added. Purple precipitate started to appear immediately, and the reaction was stirred under the argon atmosphere at room temperature for 24 hours. The resulting clear purple solution was filtered off and 2 mL of

it was sealed in a 10 cm long ampule under argon atmosphere and placed into a –10 ºC freezer. After 3 days, block-shaped, purple crystals of **1m** were crystallized on the walls of the ampule.

{$[Cr(acac)_3NaCr(acac)_3]^+[Fe(hfac)_3NaFe(hfac)_3]^-$} (1i)

A 50 mL Schlenk flask was charged with a mixture of $[Cr(acac)_3]$ (0.025 g, 0.071 mmol) and $[NaFe(hfac)_3]$ (0.050 g, 0.071 mmol) under a dry argon atmosphere, and 30 mL of dry, oxygen-free hexanes was added. Purple precipitate appeared immediately, and the reaction was stirred under argon atmosphere at room temperature for 24 hours. The solvent was then removed by vacuum and the purple reaction residue was dried under vacuum at 50-60 ºC sand bath overnight. The solid residue was then sealed in 10 cm long evacuated ampule and placed in a gradient furnace at 90 ºC with a temperature difference of 10 ºC. Molecular isomer **1m** was found to sublime into the cold zone of the container after 1 week, while the ionic isomer **1i** remained in the hot zone. The yield was *ca*. 0.075 g (50%). The purity of the crystalline product was confirmed by X-ray powder diffraction analysis (Figure S4 and Table S2). ICP-OES (2% HNO_3 water solution, 20 ºC): Cr, 4.94% (Calcd: 4.96%); Na, 2.15% (2.19%); Fe: 5.20% (5.32%).

3. X-Ray Powder Diffraction Patterns of 1m and 1i Isomers

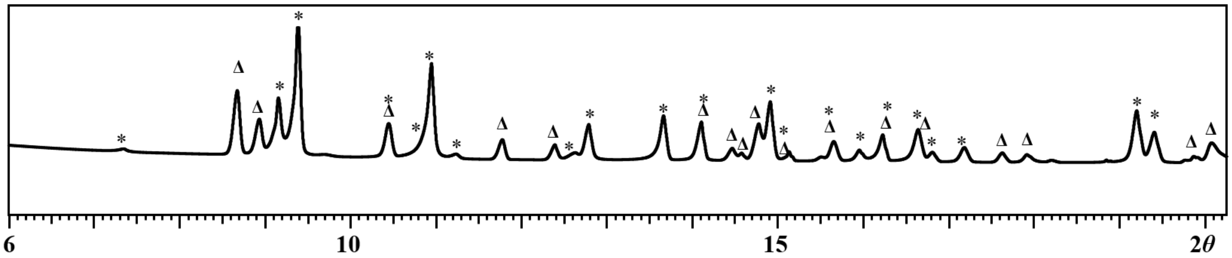

Figure S1. X-ray powder diffraction pattern of solid residue obtained upon evaporation of solvent from the reaction (1) in hexanes after 24 hours. The Δ and * labels designate **1i** and **1m** peaks, respectively, in X-ray powder diffraction pattern between 2θ = 6-20°.

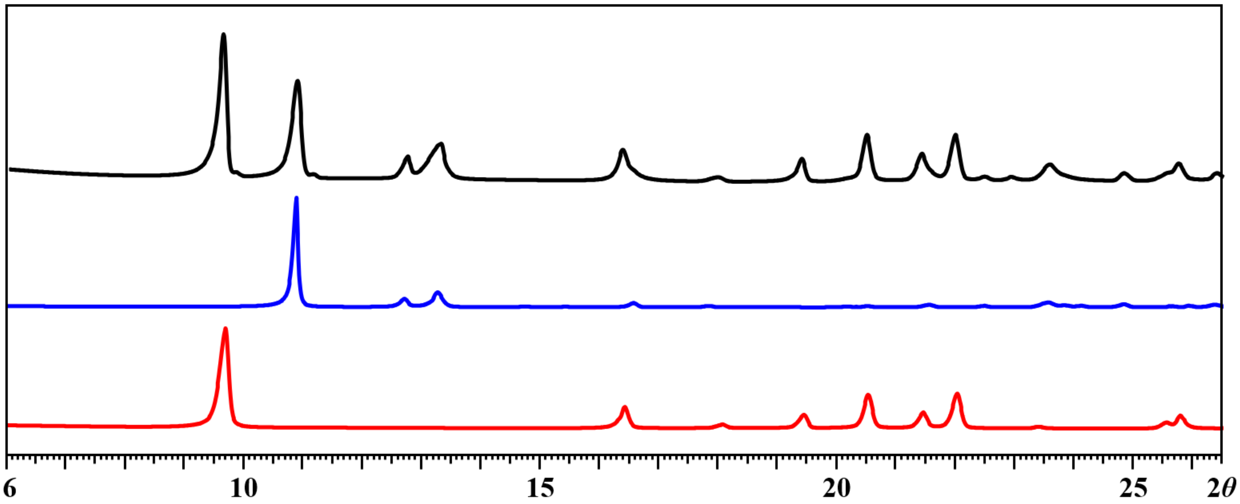

Figure S2. X-ray powder diffraction patterns of the residue obtained from evaporation of **1m** solution in acetone (black), $[Cr(acac)_3]$ (blue), and $[NaFe(hfac)_3]$ (red).

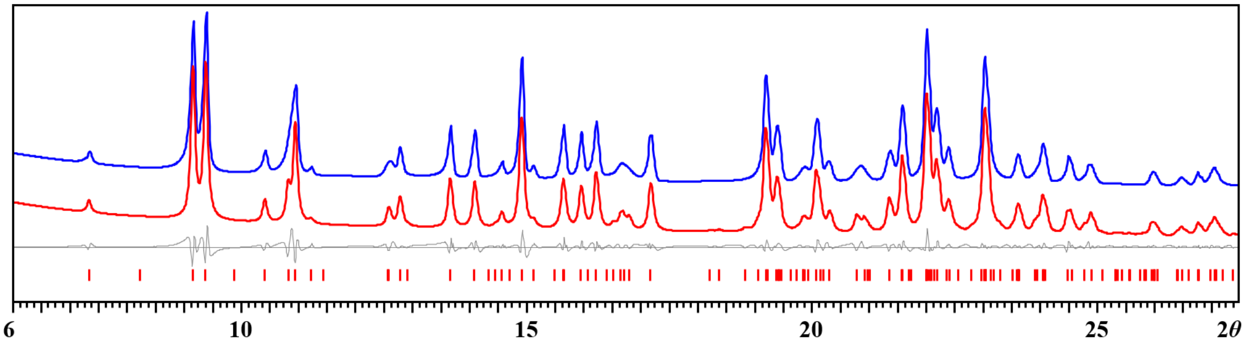

Figure S3. X-ray powder diffraction pattern and Le Bail fit for the bulk powder of **1m** isomer. Blue and red curves are experimental and calculated patterns, respectively. Gray is the difference curve. Theoretical peak positions are shown as red bars at the bottom.

Table S1. Unit Cell Parameters of **1m** Isomer Obtained by the Le Bail Fit and from the Single Crystal Data.

**1m**		
	Le Bail Fit (20 °C)	Single Crystal Data (-173 °C)
Space Group	*P*-1	*P*-1
a (Å)	9.31018(11)	9.1400(3)
b (Å)	12.52951(10)	12.2115(4)
c (Å)	19.18736(6)	18.8272(7)
α (°)	81.3505(15)	81.6939(9)
β (°)	81.0961(5)	80.9536(9)
γ (°)	75.1769(13)	76.1981(9)
V (Å^3)	2123.25(10)	2002.74(12)

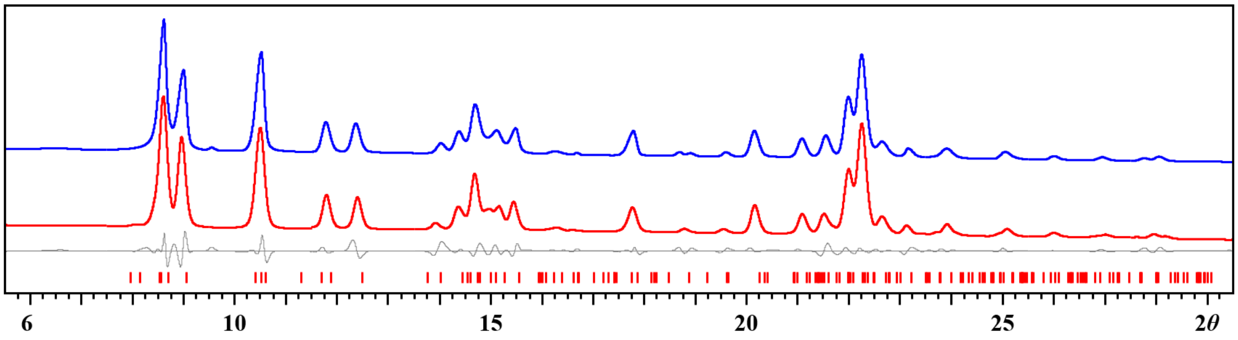

Figure S4. X-ray powder diffraction pattern and Le Bail fit for the bulk powder of **1i** isomer. Blue and red curves are experimental and calculated patterns, respectively. Gray is the difference curve. Theoretical peak positions are shown as red bars at the bottom.

Table S2. Unit Cell Parameters of **1i** Isomer Obtained by the Le Bail Fit and from the Single Crystal Data.

**1i**		
	Le Bail Fit (20 °C)	Single Crystal Data (-173 °C)
Space Group	*P*-1	*P*-1
a (Å)	11.9743(3)	11.3772(5)
b (Å)	12.4128(2)	12.1687(6)
c (Å)	17.2408(4)	17.2565(8)
α (°)	72.1073(19)	71.8810(10)
β (°)	74.5853(18)	73.7290(10)
γ (°)	62.2426(14)	64.8960(10)
V ($Å^3$)	2134.98(8)	2025.41(16)

4. Single Crystal Growth

Single crystals of **1m** isomer suitable for X-ray structural measurements were obtained by sublimation method. Bulk powder of **1m** was sealed in an evacuated ampule which was placed in a 90 ºC gradient furnace with a temperature difference of 10 ºC. Purple, block-shaped crystals of **1m** were deposited in the cold zone of the ampule after 2 days.

Single crystals of **1i** isomer suitable for X-ray structural measurements were obtained by low temperature solution method. The hexanes solution for crystal growth was taken from the synthesis of **1i** isomer as described in the Section. After filtering off all purple precipitate at room temperature, the solution was sealed in an ampule under argon and was placed in -15 ºC freezer. Purple, block-shaped crystals of **1i** were crystallized on the wall of container in 2-3 days.

5. X-Ray Crystallographic Procedures

Single Crystal X-Ray Diffraction Procedures for 1m and 1i Isomers

Single crystal diffraction data for **1m** and **1i** isomers were measured at 100(2) K on a Huber Kappa 4-Circle diffraction system with a DECTRIS PILATUS3 X 2M (CdTe) pixel array detector using ϕ scans (synchrotron radiation at λ = 0.41328 Å) located at the Advanced Photon Source, Argonne National Laboratory (NSF ChemMatCARS, Sector 15, Beamline 15-ID-D). Diffraction data were collected with 0.5 s frames using ϕ scans while manually attenuating the beam to minimize overages of individual pixels. Data reduction and integration were performed with the Bruker software package SAINT (version 8.38A).[4] Data were corrected for absorption effects using the empirical methods as implemented in SADABS (version 2016/2).[5] The structures were solved by SHELXT (version 2018/2)[6] and refined by full-matrix least-squares procedures using the SHELXL (version 2019/2)[7] software package. All non-hydrogen atoms were refined anisotropically. Hydrogen atoms were included in idealized positions for structure factor calculations, with U_{iso}(H) = 1.2 U_{eq}(C) and U_{iso}(H) = 1.5 U_{eq}(C) for methyl groups. Three CF_3 groups in **1i** were found to be rotationally disordered. All disordered parts were modeled with anisotropic thermal parameters using similarity restraints. The displacement parameters of disordered parts were also restrained with the combination of RIGU/SIMU commands. All restraint commands were applied using their program default estimated standard deviations, except the deviation of 0.01 applied for SIMU. DFIX and SADI instructions used in order to make C–C and C–F bond distances chemically reasonable. Crystallographic data, details of the data collection and structure refinement for these structures are listed in Table S3.

Synchrotron X-Ray Resonant Diffraction Procedures

Single crystals of heterotrimetallic compound **1m** and **1i** were mounted on a glass fiber and cooled to 100(2) K using an Oxford Instruments Cryojet cryostat. The Huber Kappa 4-Circle diffraction system, integrated with a DECTRIS PILATUS3 X 2M(CdTe) pixel array detector, was modified for synchrotron use at the ChemMatCARS 15-ID-D beamline at the Advanced Photon Source (Argonne National Laboratory). For **1m**, diffraction data were collected at three different energies: 30 keV, away from both absorption edges, 7.091 keV, which is slightly below the Fe *K*-edge, and 5.966 keV, which is slightly below the Cr *K*-edge using ϕ scans while manually attenuating the beam to minimize overages of individual pixels. For **1i**, diffraction data were collected at three different energies: 30, keV away from both absorption edges, 7.084 keV, which is slightly below the Fe *K*-edge, and 5.965 keV, which is slightly below the Cr *K*-Edge using ϕ scans while manually attenuating the beam to minimize overages of individual pixels. Data reduction and integration were performed with the Bruker APEX3 software package.[4] Data were scaled and corrected for absorption effects using the multi-scan procedure as implemented in SADABS.[5] The structure was solved by SHELXT[6] and refined by a full-matrix least-squares procedure using the Bruker SHELXL (version 2019/2)[7] software package through the OLEX2 graphical interface.[8] The scan at 30 keV, which is energetically well above the atomic absorption energies, gave a least-squares

refinement of all model positional and displacement parameters. Crystallographic data and details of the data collection and structure refinement are listed in Table S2.

For further refinement of the Cr/Fe site compositions, anomalous data sets collected at the lower-energy side of the absorption edges were used to minimize the solid-state effects neglected for calculations of dispersion factors.[9-12] The corresponding occupancies were refined using OLEX2.[8] The resonant scattering factors are calculated according to Cromer & Liberman algorithm.[13] This method has the benefit of reporting refined values with standard uncertainties and provides a straightforward scaling of the different anomalous data sets. The converged positional and displacement parameters of the final model from the 30 keV data were utilized and kept fixed while the Cr/Fe occupancies of the transition metal sites were refined. Difference Fourier electron density maps at the Cr and Fe *K*-edges were obtained by generating structure factor files without least-square refinements of the atomic model generated by the data sets at 30 keV with the reflection data obtained at the respective metal absorption edges. The maps were visualized with the program OLEX2.[8] Simultaneous least-square refinement of the Cr and Fe sites against several data sets collected at different wavelengths near-edge of the anomalous scatters provided occupancies of 0.997(9) for Cr site and 1.010(10) for the Fe site in **1m** and 1.009(10) for Cr site and 1.033(11) for the Fe site in **1i**.

X-Ray Fluorescence Spectroscopy Procedures

Single crystals of **1m** and **1i** were mounted on a glass fiber and cooled to 100(2) K using an Oxford Instruments Cryojet cryostat. The beam energy was lowered to the Cr *K*-edge (5989 eV) and Fe *K*-edge (7112 eV), respectively. X-ray fluorescence scans were collected in a step of 1 eV by using a Vortex-60EX X-ray fluorescence detector. The X-ray fluorescence scans of pure crystalline powders of [$Cr^{III}(acac)_3$] as Cr^{3+} standard, [$NaFe^{II}(ptac)_3$] as Fe^{2+} standard, and $Fe^{III}(hfac)_3$ as Fe^{3+} standard were collected similarly around the Cr *K*-edge and Fe *K*-edge, separately. All collected spectra were referenced against the *K*-edge of Cu-foil (8979 eV). The *K*-edge values in different compounds spectra were extracted from the first order derivative of the near edge spectra. The standard deviation for measuring all *K*-edge energy values is 10^{-4} eV.

Table S3. Crystal Data and Structure Refinement Parameters for **1m** and **1i**

**Compound**	**1m**	**1i**
Empirical formula	$C_{30}H_{24}CrF_{18}FeNaO_{12}$	$C_{60}H_{48}Cr_2F_{36}Fe_2Na_2O_{24}$
CCDC Number	2354168	2354169
Formula weight	1049.33	2098.66
Temperature (K)	100(2)	100(2)
Wavelength (Å)	0.41328	0.41328
Crystal system	Triclinic	Triclinic
Space group	*P*-1	*P*-1
a (Å)	9.1400(3)	11.3772(5)
b (Å)	12.2115(4)	12.1687(6)
c (Å)	18.8272(7)	17.2565(8)
α (°)	81.6939(9)	71.8810(10)
β (°)	80.9536(9)	73.7290(10)
γ (°)	76.1981(9)	64.8960(10)
V (Å^3)	2002.74(12)	2025.41(16)
Z	2	1
ρ_{calcd} (g·cm^{-3})	1.740	1.721
μ (mm^{-1})	0.188	0.186
F(000)	1046	1046
Crystal size (mm)	0.05×0.06×0.06	0.03×0.05×0.07
θ range for data collection (°)	1.130-19.836	1.167-17.120
Reflections collected	113350	67927
Independent reflections	17916 [R_{int} = 0.0511]	11082 [R_{int} = 0.0798]
Transmission factors (min/max)	0.6312/0.7053	0.5532/0.7307
Data/restraints/params.	17916/0/574	11082/348/655
$R1$,[a] $wR2$[b] ($I > 2\sigma(I)$)	0.0342, 0.0993	0.0506, 0.1203
$R1$,[a] $wR2$[b] (all data)	0.0417, 0.1026	0.0736, 0.1329
Quality-of-fit[c]	1.088	1.024

[a] $R1 = \Sigma||F_o|-|F_c||/\Sigma|F_o|$. [b] $wR2 = [\Sigma[w(F_o^2-F_c^2)^2]/\Sigma[w(F_o^2)^2]]$.

[c] Quality-of-fit = $[\Sigma[w(F_o^2-F_c^2)^2]/(N_{obs}-N_{params})]^{½}$, based on all data

6. X-Ray Single Crystal Refinement of Different Assignments of Fe and Cr Site Occupancy in 1m and 1i Isomers

Table S4. Refinement Results for Different Assignments of Fe and Cr Site Occupancy in **1m**.

**1m**	[Cr(acac)$_3$-Na-Fe(hfac)$_3$]	[Fe(acac)$_3$-Na-Cr(hfac)$_3$]	[Cr$_{0.5}$Fe$_{0.5}$(acac)$_3$-Na-Cr$_{0.5}$Fe$_{0.5}$(hfac)$_3$]
R_1,[a]	5.10%,	6.25%	5.46%,
wR_2[b] (all data)	9.13%	11.89%	10.92%
Quality-of-fit[c]	1.040	1.355	1.052
Largest diff. peak/ hole (ē·Å^{-3}) around [M(hfac)$_3$] unit	-0.41	0.96	0.52
Largest diff. peak/ hole (ē·Å^{-3}) around [M(acac)$_3$] unit	0	-1.24	-0.80

[a]$R1 = \Sigma||F_o|-|F_c||/\Sigma|F_o|$. [b]$wR2 = [\Sigma[w(F_o^2-F_c^2)^2]/\Sigma[w(F_o^2)^2]]$.
[c]Quality-of-fit = $[\Sigma[w(F_o^2-F_c^2)^2]/(N_{obs}-N_{params})]^{½}$, based on all data.

Table S5. Refinement Results for Different Assignments of Fe and Cr Site Occupancy in **1i**.

**1i**	[Cr(acac)$_3$-Na-Cr(acac)$_3$][Fe(hfac)$_3$-Na-Fe(hfac)$_3$]	[Fe(acac)$_3$-Na-Fe(acac)$_3$][Cr(hfac)$_3$-Na-Cr(hfac)$_3$]	[Cr(acac)$_3$-Na-Fe(acac)$_3$][Cr(hfac)$_3$-Na-Fe(hfac)$_3$]
R_1,[a]	5.33%	6.45%	5.70%
wR_2[b] (all data)	10.68%	13.35%	12.48%
Quality-of-fit[c]	1.059	1.326	1.078
Largest diff. peak/ hole (ē·Å^{-3}) around [M(hfac)$_3$] units	0	1.11	0.71
Largest diff. peak/ hole (ē·Å^{-3}) around [M(acac)$_3$] units	-0.63	-1.36	-0.90

[a]$R1 = \Sigma||F_o|-|F_c||/\Sigma|F_o|$. [b]$wR2 = [\Sigma[w(F_o^2-F_c^2)^2]/\Sigma[w(F_o^2)^2]]$.
[c]Quality-of-fit = $[\Sigma[w(F_o^2-F_c^2)^2]/(N_{obs}-N_{params})]^{½}$, based on all data.

7. Solid State Structures of Molecular and Ionic Isomers

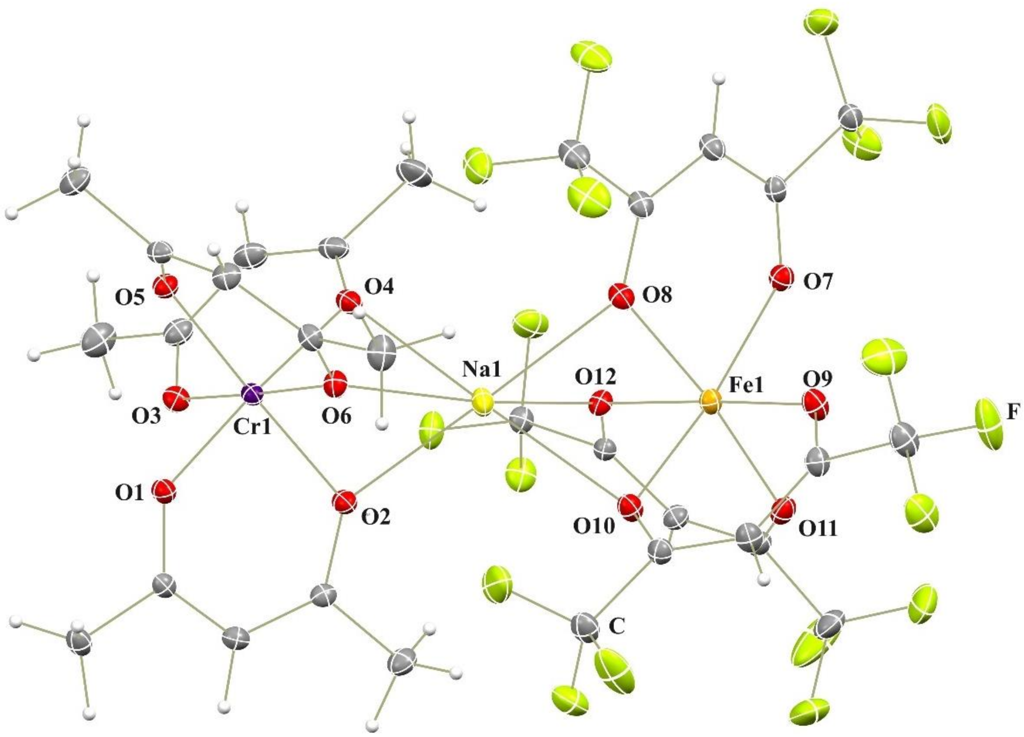

Figure S5. Solid-state structure of **1m** isomer drawn with thermal ellipsoids at the 30% probability level. Hydrogen atoms are represented by spheres of arbitrary radius.

Table S6. Bond Distances (Å) and Angles (deg.) in the Structure of **1m** Isomer.

**Bond distances**					
Cr(1)–O(1)	1.9418(7)	Fe(1)–O(7)	2.0822(8)	Na(1)–O(2)	2.3933(9)
Cr(1)–O(2)	1.9708(7)	Fe(1)–O(8)	2.0978(8)	Na(1)–O(4)	2.4429(9)
Cr(1)–O(3)	1.9575(8)	Fe(1)–O(9)	2.0630(8)	Na(1)–O(6)	2.4234(9)
Cr(1)–O(4)	1.9603(8)	Fe(1)–O(10)	2.0933(8)	Na(1)–O(8)	2.3944(9)
Cr(1)–O(5)	1.9471(7)	Fe(1)–O(11)	2.0655(8)	Na(1)–O(10)	2.5075(9)
Cr(1)–O(6)	1.9604(7)	Fe(1)–O(12)	2.0711(7)	Na(1)–O(12)	2.4860(9)
**Angles**					
O(1)–Cr(1)–O(2)	90.91(3)	O(7)–Fe(1)–O(8)	85.88(3)	O(2)–Na(1)–O(4)	67.36(3)
O(1)–Cr(1)–O(3)	91.70(3)	O(7)–Fe(1)–O(10)	169.92(3)	O(2)–Na(1)–O(6)	67.47(3)
O(1)–Cr(1)–O(4)	176.23(3)	O(9)–Fe(1)–O(7)	90.57(3)	O(2)–Na(1)–O(8)	174.76(3)
O(1)–Cr(1)–O(5)	89.91(3)	O(9)–Fe(1)–O(8)	92.41(3)	O(2)–Na(1)–O(10)	111.93(3)
O(1)–Cr(1)–O(6)	91.97(3)	O(9)–Fe(1)–O(10)	86.85(3)	O(2)–Na(1)–O(12)	103.88(3)
O(3)–Cr(1)–O(2)	93.47(3)	O(9)–Fe(1)–O(11)	93.46(3)	O(4)–Na(1)–O(10)	170.24(3)
O(3)–Cr(1)–O(4)	90.71(3)	O(9)–Fe(1)–O(12)	176.72(3)	O(4)–Na(1)–O(12)	98.34(3)
O(3)–Cr(1)–O(6)	176.27(3)	O(10)–Fe(1)–O(8)	84.50(3)	O(6)–Na(1)–O(4)	66.37(3)
O(4)–Cr(1)–O(2)	86.05(3)	O(11)–Fe(1)–O(7)	97.44(3)	O(6)–Na(1)–O(10)	122.88(3)
O(5)–Cr(1)–O(2)	177.56(3)	O(11)–Fe(1)–O(8)	173.22(3)	O(6)–Na(1)–O(12)	164.22(3)
O(5)–Cr(1)–O(3)	88.79(3)	O(11)–Fe(1)–O(10)	92.44(3)	O(8)–Na(1)–O(4)	109.73(3)
O(5)–Cr(1)–O(4)	93.04(3)	O(11)–Fe(1)–O(12)	87.06(3)	O(8)–Na(1)–O(6)	115.83(3)
O(5)–Cr(1)–O(6)	91.91(3)	O(12)–Fe(1)–O(7)	92.58(3)	O(8)–Na(1)–O(10)	70.14(3)
O(6)–Cr(1)–O(2)	85.77(3)	O(12)–Fe(1)–O(8)	86.88(3)	O(8)–Na(1)–O(12)	71.91(3)
O(6)–Cr(1)–O(4)	85.59(3)	O(12)–Fe(1)–O(10)	89.89(3)	O(12)–Na(1)–O(10)	72.19(3)

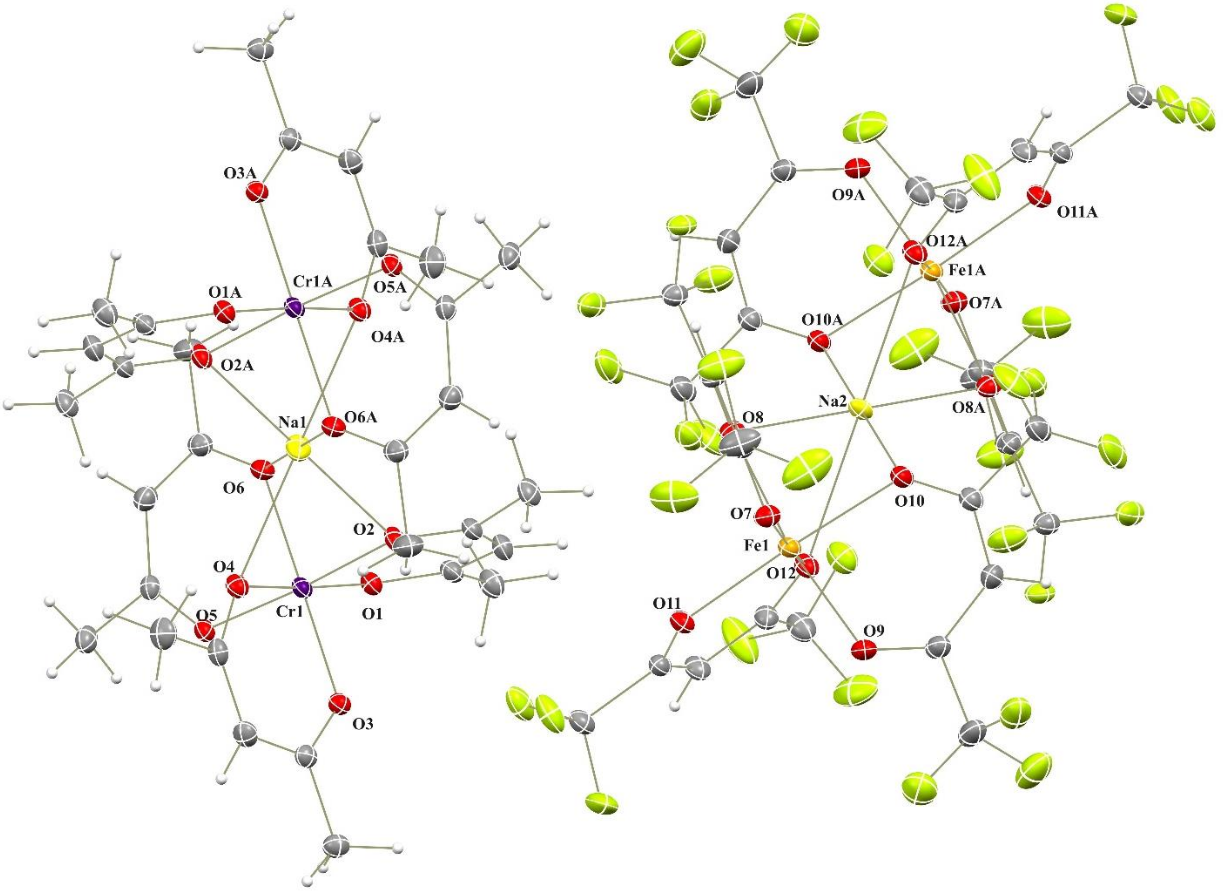

Figure S6. Solid state structure of **1i** isomer drawn with thermal ellipsoids at the 30% probability level. Hydrogen atoms are represented by spheres of arbitrary radius.

Table S7. Bond Distances (Å) and Angles (deg.) in the Structure of **1i** Isomer.

**Bond distances**					
Cr(1)–O(1)	1.9467(17)	Fe(1)–O(7)	2.0746(18)	Na(1)–O(2)	2.2925(19)
Cr(1)–O(2)	1.9573(16)	Fe(1)–O(8)	2.080(2)	Na(1)–O(4)	2.3959(17)
Cr(1)–O(3)	1.9428(19)	Fe(1)–O(9)	2.064(2)	Na(1)–O(6)	2.3586(19)
Cr(1)–O(4)	1.9626(17)	Fe(1)–O(10)	2.0845(17)	Na(2)–O(8)	2.6395(19)
Cr(1)–O(5)	1.9495(16)	Fe(1)–O(11)	2.0600(16)	Na(2)–O(10)	2.456(2)
Cr(1)–O(6)	1.978(2)	Fe(1)–O(12)	2.0902(18)	Na(2)–O(12)	2.6031(18)
**Angles**					
O(1)–Cr(1)–O(2)	90.78(7)	O(7)–Fe(1)–O(8)	86.56(8)	O(2)–Na(1)–O(2A)	180.0
O(1)–Cr(1)–O(4)	176.01(7)	O(7)–Fe(1)–O(10)	91.81(7)	O(2)–Na(1)–O(4A)	110.91(6)
O(1)–Cr(1)–O(5)	91.54(7)	O(7)–Fe(1)–O(12)	173.72(8)	O(2)–Na(1)–O(4)	69.08(6)
O(1)–Cr(1)–O(6)	91.99(8)	O(8)–Fe(1)–O(10)	84.34(8)	O(2)–Na(1)–O(6)	70.70(7)
O(2)–Cr(1)–O(4)	85.46(7)	O(8)–Fe(1)–O(12)	87.17(8)	O(2)–Na(1)–O(6A)	109.30(7)
O(2)–Cr(1)–O(6)	86.32(8)	O(9)–Fe(1)–O(7)	91.42(8)	O(4)–Na(1)–O(4A)	180.0
O(3)–Cr(1)–O(1)	89.97(8)	O(9)–Fe(1)–O(8)	170.50(7)	O(6)–Na(1)–O(4A)	110.86(6)
O(3)–Cr(1)–O(2)	92.92(8)	O(9)–Fe(1)–O(10)	86.45(8)	O(6)–Na(1)–O(4)	69.14(6)
O(3)–Cr(1)–O(4)	91.57(8)	O(9)–Fe(1)–O(12)	94.72(8)	O(6)–Na(1)–O(6A)	180.0
O(3)–Cr(1)–O(5)	90.13(8)	O(10)–Fe(1)–O(12)	87.27(7)	O(8)–Na(2)–O(8A)	180.0
O(3)–Cr(1)–O(6)	177.90(8)	O(11)–Fe(1)–O(7)	94.30(7)	O(10)–Na(2)–O(8A)	113.56(6)
O(3)–Cr(1)–O(6)	86.42(8)	O(11)–Fe(1)–O(8)	97.03(8)	O(10)–Na(2)–O(8)	66.44(6)
O(4)–Cr(1)–O(2)	176.17(8)	O(11)–Fe(1)–O(9)	92.37(8)	O(10)–Na(2)–O(10A)	180.0
O(5)–Cr(1)–O(4)	92.14(7)	O(11)–Fe(1)–O(10)	173.81(7)	O(10)–Na(2)–O(12A)	110.65(6)
O(5)–Cr(1)–O(6)	90.56(8)	O(11)–Fe(1)–O(12)	86.77(7)	O(10)–Na(2)–O(12)	69.35(6)
				O(12)–Na(2)–O(8A)	113.49(6)
				O(12)–Na(2)–O(8)	66.51(6)
				O(12)–Na(2)–O(12)	180.0

8. Direct Analysis in Real Time (DART) Mass Spectra of 1m and 1i Isomers

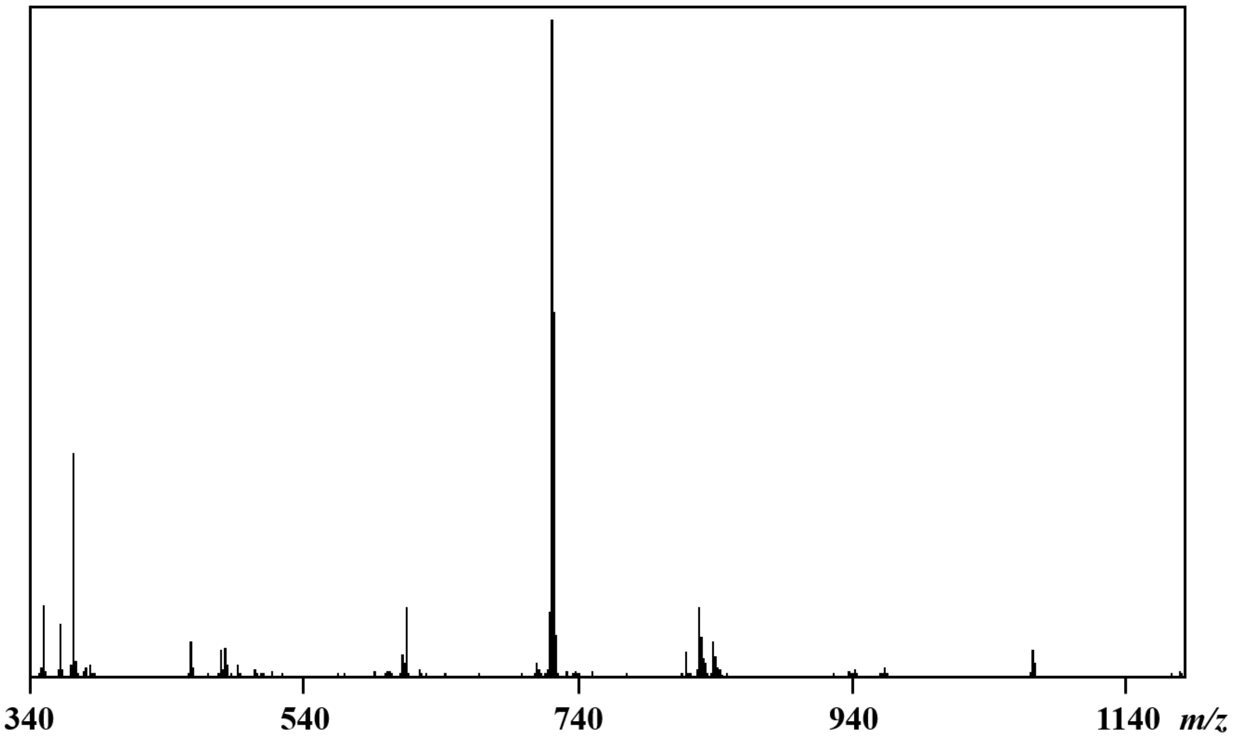

Figure S7. Positive-mode DART mass spectrum of **1m**.

Table S8. Assignment of Ions Detected in Positive-Ion DART Mass Spectrum of **1m** Isomer.

Ions	*Observed*	*Calculated*	*Δ*	*% Base*
$[Na_2FeCr(acac)_3(hfac)_3]^+$	1071.959	1071.953	0.006	5.0
$[NaFeCr(acac)_3(hfac)_2]^+$	941.980	841.975	0.005	5.1
$[NaCr_2(acac)_5(hfac)]^+$	829.090	829.082	0.008	10.4
$[NaCr_2(acac)_6]^+$	721.139	721.138	0.001	100
$[Na_2Fe(acac)(hfac)_2]^+$	614.945	614.935	0.010	10.4
$[Na_3(hfac)_2]^+$	483.955	483.953	0.002	4.4
$[HCr(acac)_2(hfac)]^+$	458.036	458.026	0.010	5.2
$[NaCr(acac)_3]^+$	372.070	372.064	0.006	34.0
$[HCr(acac)_3]^+$	350.090	350.082	0.008	10.7

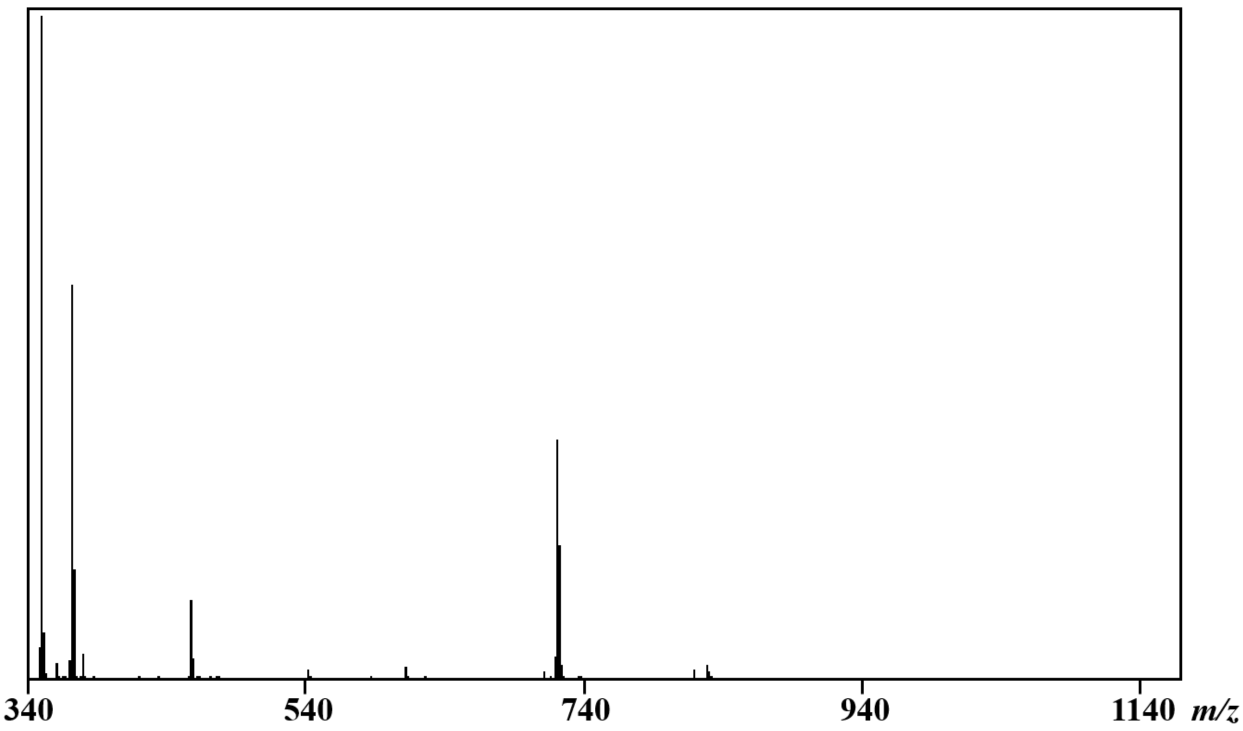

Figure S8. Positive-mode DART mass spectrum of **1i**.

Table S9. Assignment of Ions Detected in Positive-Ion DART Mass Spectrum of **1i** Isomer.

Ions	*Observed*	*Calculated*	*Δ*	*% Base*
**$[NaCr_2(acac)_6]^+$**	721.140	721.138	0.002	36.3
**$[HCr(acac)_2(hfac)]^+$**	458.038	458.026	0.012	12.2
**$[NaCr(acac)_3]^+$**	372.072	372.064	0.008	59.7
**$[HCr(acac)_3]^+$**	350.091	350.082	0.009	100

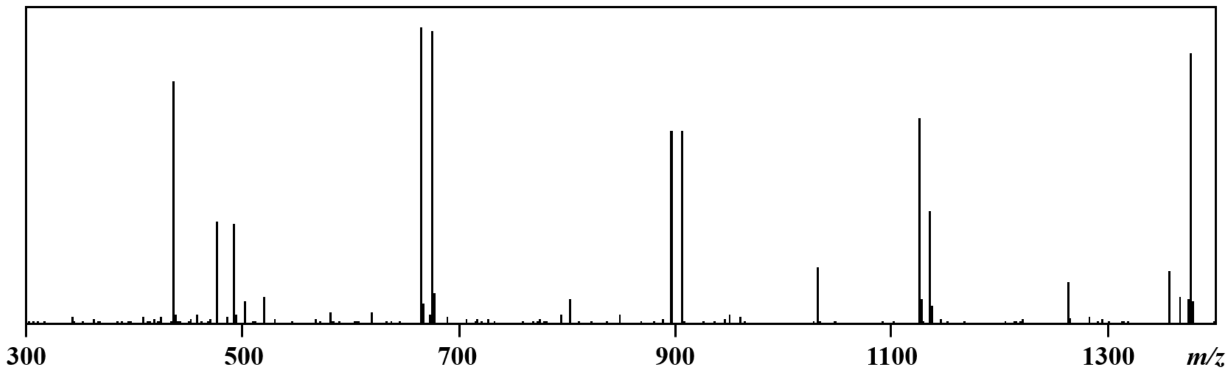

Figure S9. Negative-mode DART mass spectrum of **1m**.

Table S10. Assignment of Ions Detected in Negative-Ion DART Mass Spectrum of **1m** Isomer.

Ions	*Observed*	*Calculated*	*Δ*	*% Base*
**$[NaFe_2(hfac)_6]^-$**	1376.782	1376.788	0.006	89.2
**$[Na_5(hfac)_6]^-$**	1357.240	1357.254	0.012	21.0
**$[NaCrFe(acac)_3(hfac)_4]^-$**	1262.889	1262.906	0.017	20.2
**$[Na_2Fe(hfac)_5]^-$**	1137.059	1137.079	0.020	49.5
**$[Na_4(hfac)_5]^-$**	1126.868	1126.899	0.031	72.5
**$[CrFe(acac)_3(hfac)_3]^-$**	1032.866	1032.881	0.025	27.1
**$[NaFe(hfac)_4]^-$**	906.870	906.882	0.012	69.9
**$[Na_3(hfac)_4]^-$**	896.915	896.921	0.006	70.1
**$[Fe(hfac)_3]^-$**	676.890	676.899	0.009	98.0
**$[Na_2(hfac)_3]^-$**	666.930	666.944	0.014	100
**$[Na(hfac)_2]^-$**	436.951	436.966	0.015	79.8

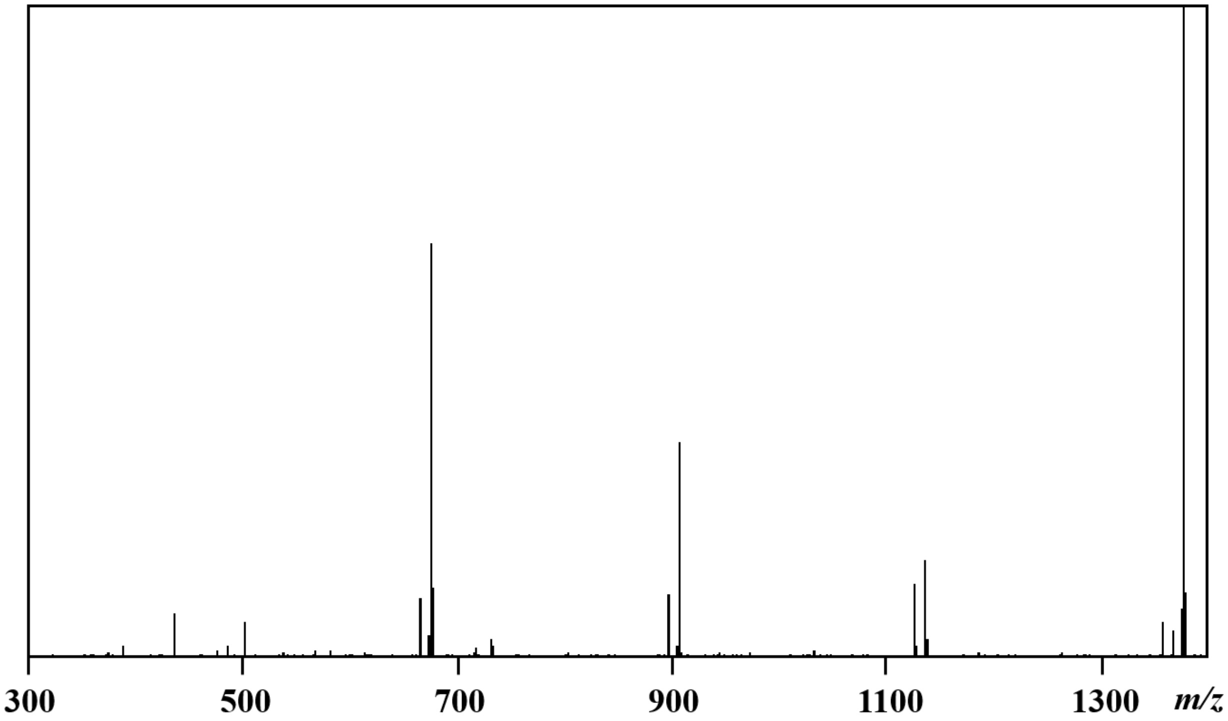

Figure S10. Negative-mode DART mass spectrum of **1i**.

Table S11. Assignment of Ions Detected in Negative-Ion DART Mass Spectrum of **1i** Isomer.

Ions	*Observed*	*Calculated*	*Δ*	*% Base*
**$[NaFe_2(hfac)_6]^-$**	1376.759	1376.788	0.029	100
**$[Na_5(hfac)_6]^-$**	1357.231	1357.254	0.023	6.2
**$[Na_2Fe(hfac)_5]^-$**	1137.050	1137.079	0.029	15.9
**$[Na_4(hfac)_5]^-$**	1126.854	1126.899	0.045	12.1
**$[NaFe(hfac)_4]^-$**	906.851	906.882	0.031	33.7
**$[Na_3(hfac)_4]^-$**	896.904	896.921	0.017	11.6
**$[Fe(hfac)_3]^-$**	676.871	676.899	0.028	63.7
**$[Na_2(hfac)_3]^-$**	666.921	666.944	0.023	10.1
**$[Na(hfac)_2]^-$**	436.940	436.966	0.026	7.5

9. Thermal Decomposition Patterns of 1m and 1i Isomers

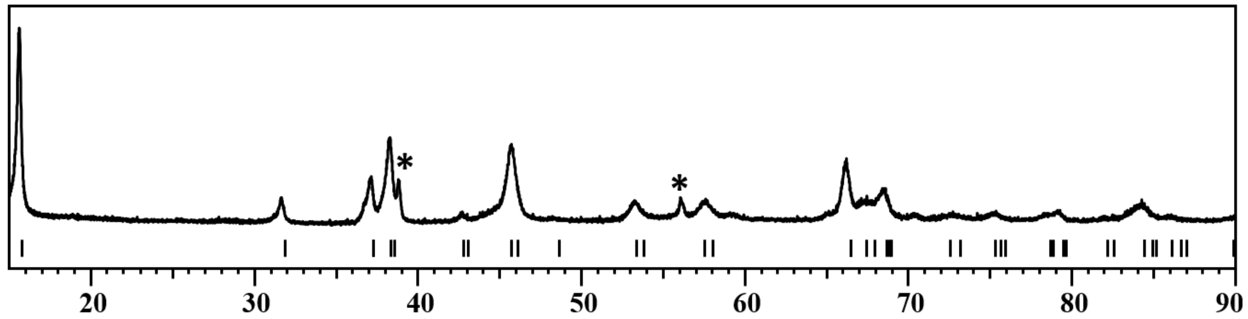

Figure S11. Powder X-ray diffraction pattern of decomposition residue of **1m** isomer at 600 °C. Peak positions of $Na_{0.5}FeO_2$ (PDF-2: No. 01-082-1496) are shown as black bars at the bottom. Peaks of impurity phase Fe_3O_4 are marked with asterisk.

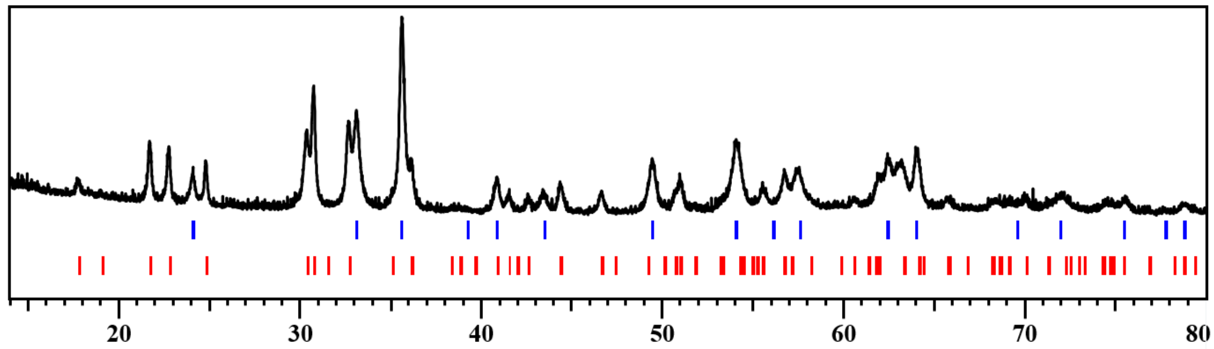

Figure S12. Powder X-ray diffraction pattern of decomposition residue of **1i** isomer at 600 °C. Peak positions of Fe_2O_3 (PDF-2: No. 00-039-1346) and Na_2CrO_4 (PDF-2: No. 01-074-0674) are shown as blue and red bars at the bottom, respectively.

10. Synthesis, Crystallization, and Structural Analysis of Molecular [Al(acac)$_3$NaMn(hfac)$_3$] and Ionic {[Al(acac)$_3$NaAl(acac)$_3$]$^+$[Mn(hfac)$_3$]$^-$} Compounds.

Synthesis and Crystallization Procedures

Single crystals of molecular [Al(acac)$_3$NaMn(hfac)$_3$] suitable for X-ray structural measurements were obtained by sublimation method. Stoichiometric amounts of [Al(acac)$_3$] (0.038 g, 0.117 mmol), anhydrous $MnCl_2$ (0.015 g, 0.119 mmol) and Na(hfac) (0.081 g, 0.354 mmol) (Reaction S1) were grinded in glovebox under argon atmosphere and sealed in 10 cm long evacuated ampule. The ampule was placed in a gradient furnace at 100 ºC with a temperature difference of *ca.* 10 ºC. Yellow, block-shaped crystals [Al(acac)$_3$NaMn(hfac)$_3$] were crystallized at the cold zone of the container.

Al(acac)$_3$ + $MnCl_2$ + 3Na(hfac) = Al(acac)$_3$NaMn(hfac)$_3$ + 2NaCl (S1)

Single crystals of ionic {[Al(acac)$_3$NaAl(acac)$_3$]$^+$[Mn(hfac)$_3$]$^-$} suitable for X-ray structural measurements were obtained by crystallization from solution at low temperature. Stoichiometric amounts of [Al(acac)$_3$] (0.077 g, 0.238 mmol), anhydrous $MnCl_2$ (0.015 g, 0.119 mmol) and Na(hfac) (0.081 g, 0.354 mmol) (Reaction S2) were added into a Schlenk flask with 8 mL oxygen- and moisture-free hexanes under an argon atmosphere. The solution was stirred at room temperature for 24 hours and the precipitate was then filtered off. 2 mL of the yellow color solution was sealed into a 10 cm ampule with argon and placed into a -10º C freezer. Yellow block-shaped crystals of [Al(acac)$_3$NaAl(acac)$_3$][Mn(hfac)$_3$] were found to crystallize on the walls of ampule after 2 days.

2Al(acac)$_3$ + $MnCl_2$ + 3Na(hfac) = [Al(acac)$_3$NaAl(acac)$_3$][Mn(hfac)$_3$] + 2NaCl (S2)

Single Crystals Measurements

The single crystal diffraction data for Al(acac)$_3$NaMn(hfac)$_3$ and [Al(acac)$_3$NaAl(acac)$_3$] [Mn(hfac)$_3$] were measured at 100(2) K on a Bruker D8 VENTURE X-ray diffractometer with PHOTON 100 CMOS detector equipped with a Mo-target fine-focus sealed X-ray tube (λ = 0.71073 Å). Data reduction and integration were performed with the Bruker software package SAINT (version 8.38A).[4] Data were corrected for absorption effects using the empirical methods as implemented in SADABS (version 2016/2).[5] The structures were solved by SHELXT (version 2018/2)[6] and refined by full-matrix least-squares procedures using the Bruker SHELXL (version 2018/3 for [Al(acac)$_3$NaMn(hfac)$_3$] and 2019/2 for [Al(acac)$_3$NaAl(acac)$_3$][Mn(hfac)$_3$]) software package.[7] All non-hydrogen atoms were refined anisotropically. Hydrogen atoms were included in idealized positions for structure factor calculations with U_{iso}(H) = 1.2 U_{eq}(C) and U_{iso}(H) = 1.5 U_{eq}(C) for methyl groups. Two CF_3 groups in the [Al(acac)$_3$NaAl(acac)$_3$][Mn(hfac)$_3$] structure were found to be rotationally disordered. The disordered parts were modeled with anisotropic thermal parameters using similarity restraints. The displacement parameters for disordered parts were also restrained with the combination of RIGU/SIMU commands. All restraint commands were applied using their default estimated standard deviations, except the deviation of 0.01 applied

for SIMU. Crystallographic data, details of the data collection and structure refinement for these structures are listed in Table S12.

Table S12. Crystal Data and Structure Refinement Parameters for [$Al(acac)_3NaMn(hfac)_3$] and [$Al(acac)_3NaAl(acac)_3$][$Mn(hfac)_3$]

**Compound**	**$Al(acac)_3NaMn(hfac)_3$**	**[$Al(acac)_3NaAl(acac)_3$] [$Mn(hfac)_3$]**
Empirical formula	$C_{30}H_{24}AlF_{18}MnNaO_{12}$	$C_{45}H_{45}Al_2F_{18}MnNaO_{18}$
CCDC Number	2354170	2354171
Formula weight	1023.40	1347.63
Temperature (K)	100(2)	100(2)
Wavelength (Å)	0.71073	0.71073
Crystal system	Triclinic	Monoclinic
Space group	*P*-1	$P2_1/c$
a (Å)	9.1144(6)	12.2977(8)
b (Å)	12.2847(8)	20.2689(14)
c (Å)	18.8027(12)	22.9681(16)
α (°)	81.725(3)	90.00
β (°)	81.490(2)	96.912(2)
γ (°)	76.157(2)	90.00
V (Å^3)	2008.7(2)	5683.4(7)
Z	2	4
ρ_{calcd} (g·cm^{-3})	1.692	1.575
μ (mm^{-1})	0.502	0.398
F(000)	1022	2732
Crystal size (mm)	0.08×0.12×0.16	0.04×0.06×0.09
θ range for data collection (°)	2.945-28.327	2.775-28.282
Reflections collected	57012	68356
Independent reflections	9929 [R_{int} = 0.0388]	13900 [R_{int} = 0.0436]
Transmission factors (min/max)	0.6587/0.7123	0.6867/0.7407
Data/restraints/params.	9929/0/574	13900/499/875
$R1$,[a] $wR2$[b] ($I > 2\sigma(I)$)	0.0440, 0.0885	0.0445, 0.0816
$R1$,[a] $wR2$[b] (all data)	0.0651, 0.0965	0.0781, 0.0923
Quality-of-fit[c]	1.064	1.023

[a]$R1 = \Sigma||F_o|-|F_c||/\Sigma|F_o|$. [b]$wR2 = [\Sigma[w(F_o^2-F_c^2)^2]/\Sigma[w(F_o^2)^2]]$.

[c]Quality-of-fit = $[\Sigma[w(F_o^2-F_c^2)^2]/(N_{obs}-N_{params})]^{½}$, based on all data

Crystal Structures of [Al(acac)$_3$NaMn(hfac)$_3$] and [Al(acac)$_3$NaAl(acac)$_3$][Mn(hfac)$_3$]

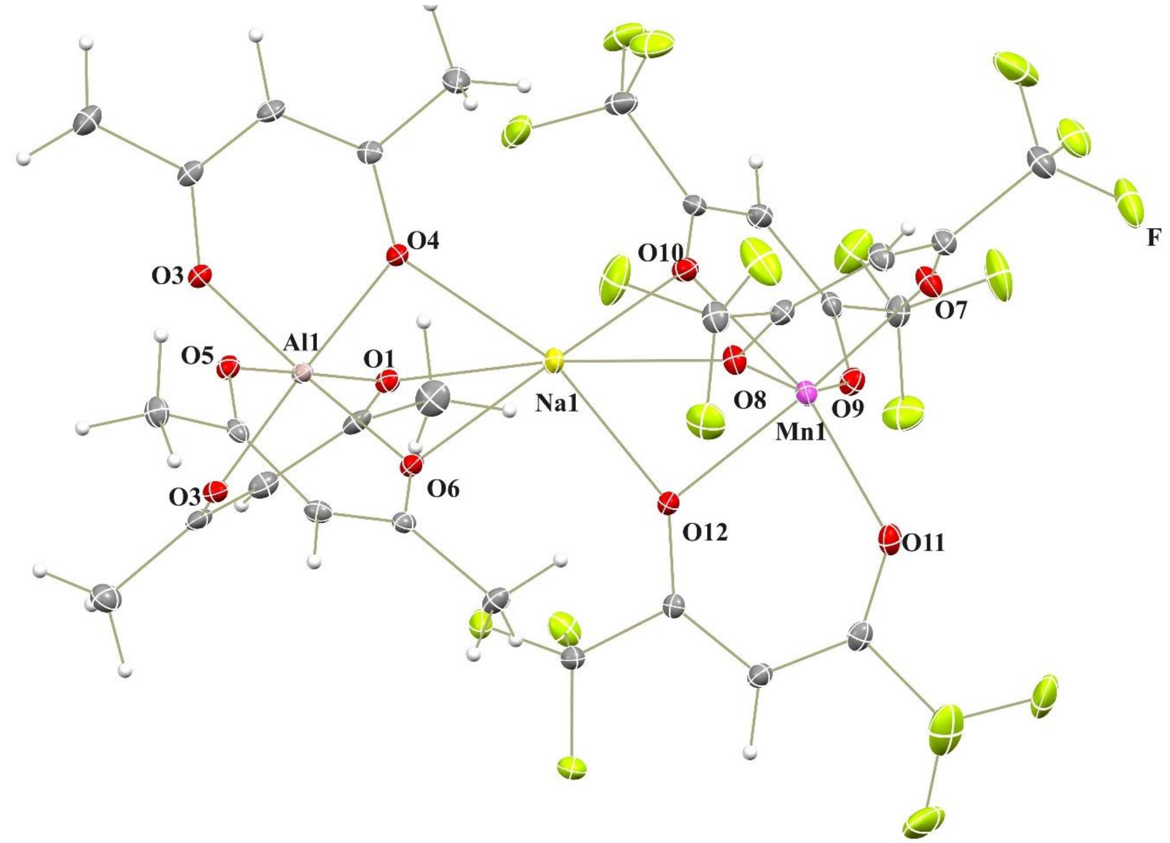

Figure S13. Solid state structure of [Al(acac)$_3$NaMn(hfac)$_3$] drawn with thermal ellipsoids at the 30% probability level. Hydrogen atoms are represented by spheres of arbitrary radius.

Table S13. Bond Distances (Å) and Angles (deg.) in the Structure of [Al(acac)$_3$NaMn(hfac)$_3$].

Bond distances					
Al(1)–O(1)	1.8765(15)	Mn(1)–O(7)	2.1101(15)	Na(1)–O(2)	2.4053(17)
Al(1)–O(2)	1.8970(15)	Mn(1)–O(8)	2.1751(15)	Na(1)–O(4)	2.4211(16)
Al(1)–O(3)	1.8637(15)	Mn(1)–O(9)	2.1235(15)	Na(1)–O(6)	2.3866(16)
Al(1)–O(4)	1.8940(15)	Mn(1)–O(10)	2.1811(15)	Na(1)–O(8)	2.4789(17)
Al(1)–O(5)	1.8523(15)	Mn(1)–O(11)	2.1288(16)	Na(1)–O(10)	2.3875(16)
Al(1)–O(6)	1.8986(15)	Mn(1)–O(12)	2.1609(14)	Na(1)–O(12)	2.4731(16)
**Angles**					
O(1)–Cr(1)–O(2)	90.17(7)	O(7)–Fe(1)–O(8)	84.05(6)	O(2)–Na(1)–O(4)	63.96(5)
O(1)–Cr(1)–O(4)	174.96(7)	O(7)–Fe(1)–O(9)	93.57(6)	O(2)–Na(1)–O(8)	172.35(6)
O(1)–Cr(1)–O(6)	93.78(7)	O(7)–Fe(1)–O(10)	96.64(6)	O(2)–Na(1)–O(12)	99.62(5)
O(2)–Cr(1)–O(6)	85.31(6)	O(7)–Fe(1)–O(11)	96.50(6)	O(4)–Na(1)–O(8)	123.61(6)
O(3)–Cr(1)–O(1)	89.20(7)	O(7)–Fe(1)–O(12)	169.27(6)	O(4)–Na(1)–O(12)	162.44(6)
O(3)–Cr(1)–O(2)	93.81(7)	O(8)–Fe(1)–O(10)	80.50(6)	O(6)–Na(1)–O(2)	64.92(5)
O(3)–Cr(1)–O(4)	91.34(7)	O(9)–Fe(1)–O(8)	162.94(6)	O(6)–Na(1)–O(4)	64.83(5)
O(3)–Cr(1)–O(6)	176.89(7)	O(9)–Fe(1)–O(10)	83.01(6)	O(6)–Na(1)–O(8)	116.06(6)
O(4)–Cr(1)–O(2)	84.79(6)	O(9)–Fe(1)–O(11)	101.45(6)	O(6)–Na(1)–O(10)	170.97(6)
O(4)–Cr(1)–O(6)	85.62(6)	O(9)–Fe(1)–O(12)	96.89(6)	O(6)–Na(1)–O(12)	103.55(6)
O(5)–Cr(1)–O(1)	91.71(7)	O(11)–Fe(1)–O(8)	95.60(6)	O(10)–Na(1)–O(2)	107.59(6)
O(5)–Cr(1)–O(2)	175.76(7)	O(11)–Fe(1)–O(10)	165.83(6)	O(10)–Na(1)–O(4)	117.41(6)
O(5)–Cr(1)–O(3)	90.02(7)	O(11)–Fe(1)–O(12)	83.76(6)	O(10)–Na(1)–O(8)	70.65(5)
O(5)–Cr(1)–O(4)	93.30(7)	O(12)–Fe(1)–O(8)	85.26(6)	O(10)–Na(1)–O(12)	72.02(5)
O(5)–Cr(1)–O(6)	90.77(6)	O(12)–Fe(1)–O(10)	82.34(5)	O(12)–Na(1)–O(8)	72.74(5)

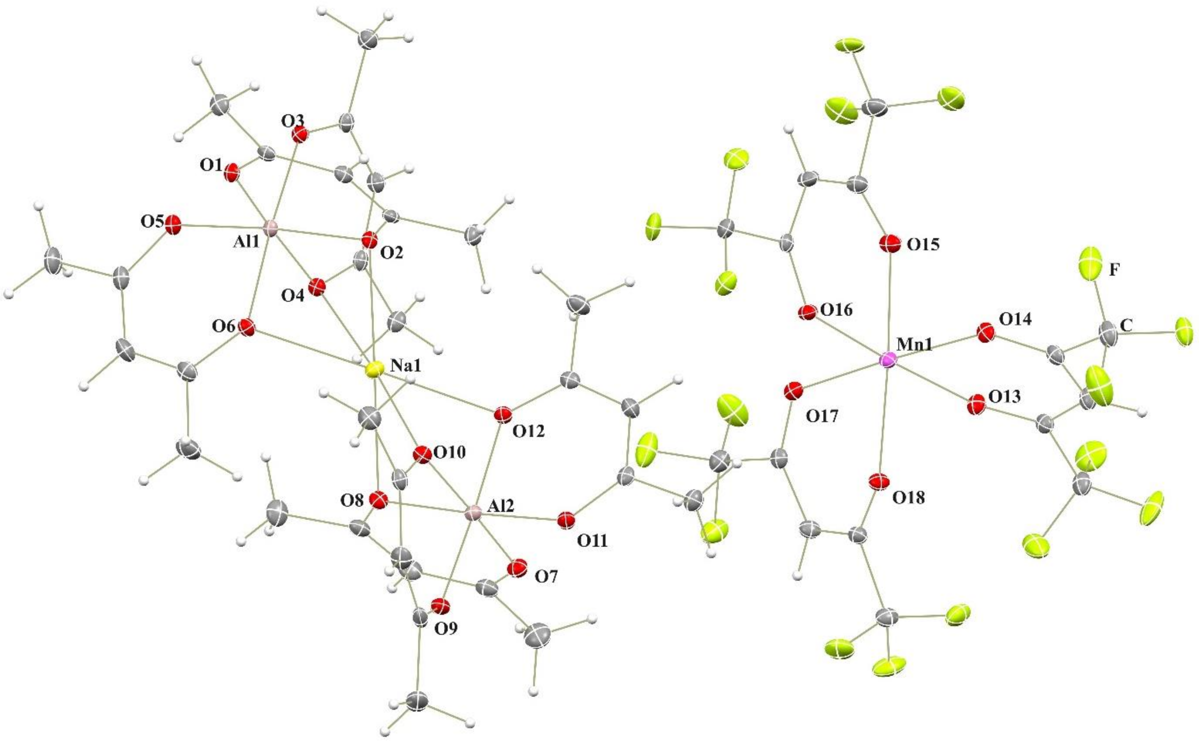

Figure S14. Solid state structure of [Al(acac)$_3$NaAl(acac)$_3$][Mn(hfac)$_3$] drawn with thermal ellipsoids at the 30% probability level. Hydrogen atoms are represented by spheres of arbitrary radius.

Table S14. Bond Distances (Å) and Angles (deg.) in the Structure of [Al(acac)$_3$NaAl(acac)$_3$][Mn(hfac)$_3$].

Bond distances							
Al(1)–O(1)	1.8687(14)	Al(2)–O(7)	1.8748(15)	Mn(1)–O(13)	2.1540(13)	Na(1)–O(2)	2.3059(15)
Al(1)–O(2)	1.9006(13)	Al(2)–O(8)	1.9032(14)	Mn(1)–O(14)	2.1505(14)	Na(1)–O(4)	2.3636(15)
Al(1)–O(3)	1.8560(14)	Al(2)–O(9)	1.8576(15)	Mn(1)–O(15)	2.1415(14)	Na(1)–O(6)	2.3666(15)
Al(1)–O(4)	1.9040(14)	Al(2)–O(10)	1.9001(14)	Mn(1)–O(16)	2.1383(13)	Na(1)–O(8)	2.3359(15)
Al(1)–O(5)	1.8705(14)	Al(2)–O(11)	1.8606(14)	Mn(1)–O(17)	2.1620(14)	Na(1)–O(10)	2.3213(15)
Al(1)–O(6)	1.8974(14)	Al(2)–O(12)	1.8973(14)	Mn(1)–O(18)	2.1675(14)	Na(1)–O(12)	2.3737(15)
**Angles**							
O(1)–Al(1)–O(2)	91.07(6)	O(7)–Al(2)–O(8)	90.73(6)	O(13)–Mn(1)–O(17)	93.04(5)	O(2)–Na(1)–O(4)	66.46(5)
O(1)–Al(1)–O(4)	175.53(6)	O(7)–Al(2)–O(10)	175.14(7)	O(13)–Mn(1)–O(18)	82.60(5)	O(2)–Na(1)–O(6)	66.73(5)
O(1)–Al(1)–O(5)	91.41(6)	O(7)–Al(2)–O(12)	92.54(6)	O(14)–Mn(1)–O(13)	83.91(5)	O(2)–Na(1)–O(8)	178.73(6)
O(1)–Al(1)–O(6)	93.63(6)	O(9)–Al(2)–O(7)	90.68(7)	O(14)–Mn(1)–O(17)	176.89(5)	O(2)–Na(1)–O(10)	114.57(5)
O(2)–Al(1)–O(4)	84.55(6)	O(9)–Al(2)–O(8)	92.62(6)	O(14)–Mn(1)–O(18)	96.19(5)	O(2)–Na(1)–O(12)	113.55(5)
O(3)–Al(1)–O(1)	89.34(6)	O(9)–Al(2)–O(10)	90.96(6)	O(15)–Mn(1)–O(13)	95.01(5)	O(4)–Na(1)–O(6)	65.85(5)
O(3)–Al(1)–O(2)	92.45(6)	O(9)–Al(2)–O(11)	89.44(6)	O(15)–Mn(1)–O(14)	90.11(6)	O(4)–Na(1)–O(12)	116.64(5)
O(3)–Al(1)–O(4)	91.75(6)	O(9)–Al(2)–O(12)	176.58(7)	O(15)–Mn(1)–O(17)	90.79(5)	O(6)–Na(1)–O(12)	177.47(6)
O(3)–Al(1)–O(5)	91.31(6)	O(10)–Al(2)–O(8)	84.63(6)	O(15)–Mn(1)–O(18)	172.97(5)	O(8)–Na(1)–O(4)	112.27(5)
O(3)–Al(1)–O(6)	176.23(6)	O(11)–Al(2)–O(7)	91.43(6)	O(16)–Mn(1)–O(13)	175.24(5)	O(8)–Na(1)–O(6)	112.88(5)
O(5)–Al(1)–O(2)	175.52(7)	O(11)–Al(2)–O(8)	177.01(7)	O(16)–Mn(1)–O(14)	91.34(5)	O(8)–Na(1)–O(12)	66.90(5)
O(5)–Al(1)–O(4)	92.90(6)	O(11)–Al(2)–O(10)	93.17(6)	O(16)–Mn(1)–O(15)	84.70(5)	O(10)–Na(1)–O(4)	176.03(6)
O(5)–Al(1)–O(6)	90.95(6)	O(11)–Al(2)–O(12)	91.65(6)	O(16)–Mn(1)–O(17)	91.71(5)	O(10)–Na(1)–O(6)	110.76(5)
O(6)–Al(1)–O(2)	85.17(6)	O(12)–Al(2)–O(8)	86.18(6)	O(16)–Mn(1)–O(18)	98.23(5)	O(10)–Na(1)–O(8)	66.70(5)
O(6)–Al(1)–O(4)	85.11(6)	O(12)–Al(2)–O(10)	85.74(6)	O(17)–Mn(1)–O(18)	82.76(5)	O(10)–Na(1)–O(12)	66.76(5)

11. References

[1] Prisecaru I. WMOSS4 Mössbauer spectral analysis software. **2009**. *www.wmoss.org*.

[2] Dennis, J. E.; Gay, D. M.; Walsh, R. E. An Adaptive Nonlinear Least-Squares Algorithm. *ACM Trans. Math. Softw.* **1981**, *7* (3), 348–368.

[3] Wei, Z.; Filatov, A. S.; Dikarev, E. V. Volatile Heterometallic Precursors for the Low-Temperature Synthesis of Prospective Sodium Ion Battery Cathode Materials. *J. Am. Chem. Soc.* **2013**, *135*, 12216–12219.

[4] SAINT; Bruker APEX3 software package: Bruker AXS, Version 2015.5-2, 2015.

[5] SADABS; part of Bruker APEX3 software package: Bruker AXS, version 2016/2, 2016.

[6] G. M. Sheldrick, *Acta Crystallogr. A* **2015**, *71*, 3-8.

[7] G. Sheldrick, *Acta Crystallogr. C* **2015**, *71*, 3-8.

[8] Dolomanov, O. V.; Bourhis, L. J.; Gildea, R. J.; Howard, J. A. K.; Puschmann, H. OLEX2: A Complete Structure Solution, Refinement and Analysis Program. *J. Appl. Crystallogr.* **2009**, *42*, 339–341.

[9] Wulf, R., Experimental Distinction of Elements with Similar Atomic Number Using Anomalous Dispersion (δ Synthesis): An Application of Synchrotron Radiation in Crystal Structure Analysis. *Acta Crystallogr. A* **1990**, *46*, 681-688.

[10] Waseda, Y., Anomalous X-Ray Scattering for Material Characterization. Springer-Verlag Berlin, Heidelberg, **2002**.

[11] Tereniak, S. J.; Carlson, R. K.; Clouston, L. J.; Young, V. G., Jr.; Bill, E.; Maurice, R.; Chen, Y. S.; Kim, H. J.; Gagliardi, L.; Lu, C. C., Role of the Metal in the Bonding and Properties of Bimetallic Complexes Involving Manganese, Iron, and Cobalt. *J. Am. Chem. Soc.* **2014**, *136*, 1842-1855.

[12] Miller, D. L.; Siedschlag, R. B.; Clouston, L. J.; Young, V. G., Jr.; Chen, Y. S.; Bill, E.; Gagliardi, L.; Lu, C. C., Redox Pairs of Diiron and Iron-Cobalt Complexes with High-Spin Ground States. *Inorg. Chem.* **2016**, *55*, 9725-9735.

[13] Cromer, D. T.; Liberman, D. A., Anomalous Dispersion Calculations near to and on the Long-Wavelength Side of an Absorption-Edge. *Acta Crystallogr. A* **1981**, *37*, 267-268.
